# Ultraviolet-A light-induced chronic hyperploidization causes fibrosis in post-mitotic cells affected by Fuchs endothelial corneal dystrophy

**DOI:** 10.1038/s41420-026-03292-8

**Published:** 2026-08-08

**Authors:** Yadav Adhikari, Mohit Parekh, Neha Deshpande, Annie Miall, Queenie Cheung, Marianne O. Price, Francis W. Price, Vicki P. Losick, Ula V. Jurkunas

**Affiliations:** 1 Schepens Eye Research Institute of Massachusetts Eye and Ear, Boston, MA USA; 2 Department of Ophthalmology, Harvard Medical School, Boston, MA USA; 3 PriceVision Group, Indianapolis, IN USA; 4 Biology Department, Boston College, Chestnut Hill, MA USA

**Keywords:** Senescence, Ageing

## Abstract

Fuchs endothelial corneal dystrophy (FECD) is a progressive disorder characterized by corneal endothelial (CE) cell loss and accumulation of extracellular matrix (ECM) excrescences (guttae) in Descemet’s membrane, leading to corneal edema and visual impairment. We previously demonstrated that ultraviolet-A (UVA) exposure induces oxidative stress and CE cell loss in a non-genetic murine model of FECD. Here, we investigated whether UVA triggers polyploidization, a conserved stress-adaptation mechanism observed in several post-mitotic tissues. Using our UVA-based FECD model, we performed temporal analyses of CE cell ploidy, and molecular markers associated with cell-cycle regulation and ECM deposition seen in FECD. UVA exposure induced oxidative DNA damage and apoptosis, resulting in decreased cell density. Surviving CE cells re-entered the cell cycle and underwent endocycling, generating enlarged polyploid nuclei in about 60% of cells with increased DNA content reaching up to 64 C by day 3, compared to 6 C at day 1 post-UVA. Early polyploidization served a compensatory role preserving tissue integrity following cell loss. However, persistent oxidative stress promoted progression toward endomitosis and multinucleation, accompanied by cellular senescence and fibrosis observed during disease progression. Mechanistically, UVA-induced cell-cycle re-entry and polyploidization were mediated by YAP1 and its downstream effectors, E2F1 and CDK2. Pharmacologic inhibition of YAP1 using the small-molecule CA3 suppressed polyploidization-associated multinucleation and reduced pro-fibrotic changes, ultimately mitigating CE cell loss in vivo. Together, these findings identify distinct early protective and later pathogenic roles of polyploidization in CE cells and highlight YAP1-mediated hyperploidization as a potential therapeutic target for FECD.

**Figure:**
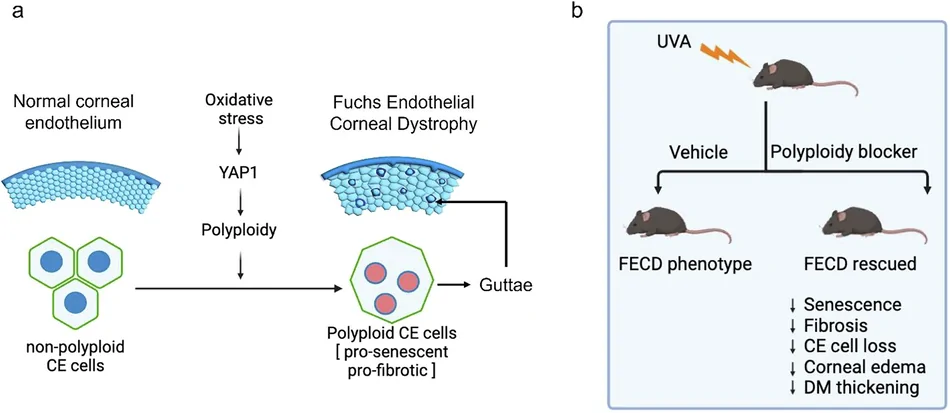
Fuchs endothelial corneal dystrophy (FECD) involves progressive corneal endothelial cell loss driven by oxidative stress. Using a UVA-induced mouse model, we show that surviving cells undergo YAP1-mediated polyploidization. While initially compensatory, persistent hyperploidization promotes senescence and disease progression. Inhibiting YAP1 reduces polyploidy and preserves endothelial cell survival, identifying a potential therapeutic target.

Fuchs endothelial corneal dystrophy (FECD) involves progressive corneal endothelial cell loss driven by oxidative stress. Using a UVA-induced mouse model, we show that surviving cells undergo YAP1-mediated polyploidization. While initially compensatory, persistent hyperploidization promotes senescence and disease progression. Inhibiting YAP1 reduces polyploidy and preserves endothelial cell survival, identifying a potential therapeutic target.

## Introduction

**Fig. 1:**
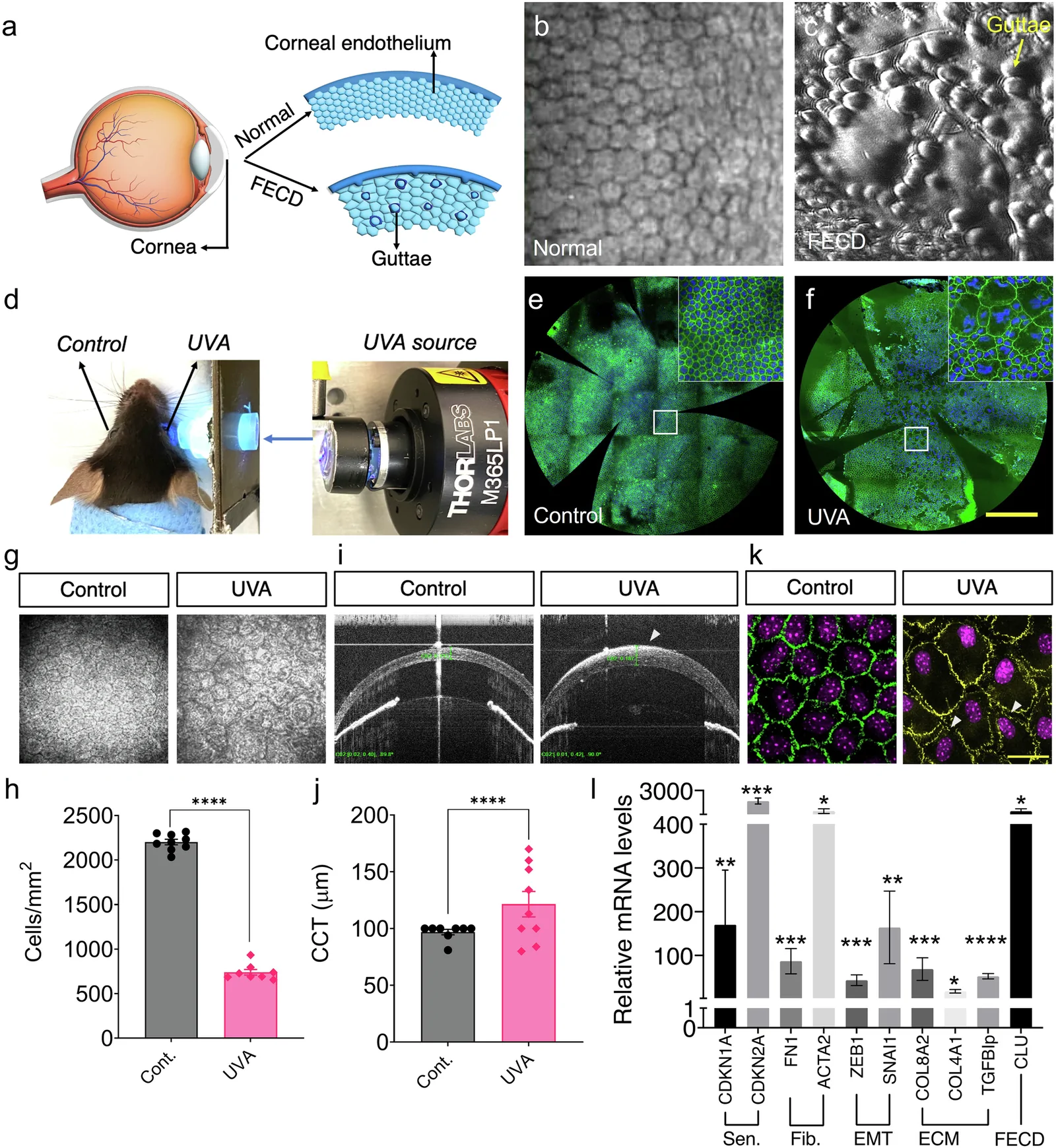
UVA based mouse model recapitulates the characteristics of FECD patients. **a** Schematic representation of the human eye illustrating the corneal endothelium under normal conditions and in FECD. FECD is characterized by a reduction in CE cell density and the presence of guttae. Representative specular microscopy images showing a regular CE cell mosaic in a healthy human eye (**b**) and CE cell loss with guttae formation (yellow arrow) in an FECD patient (**c**). **d** Experimental set up for UVA exposure in mice. 8-weeks-old female C57BL/6J mice were anaesthetized with ketamine/xylazine, wrapped in a heat retention fabric and the right eye was exposed to UVA light (500 J/cm^2^); the contralateral unexposed eye served as control. Whole-mount immunostaining for ZO-1(green) reveals normal hexagonal CE cell morphology with uniform size and density in unexposed control eyes (high magnification-inset), and marked CE cell loss, pleomorphism, and polymegathism in UVA-exposed eyes at day-14 (high magnification-inset) (**f**). Scale bar = 0.5 mm. In vivo confocal imaging (HRT) shows a reduction in CE cell density in UVA-exposed eyes (**h**) as compared to controls (**g**). Anterior segment optical coherence tomography (AS-OCT) (**i**) reveals increased central corneal thickness (CCT) in UVA-exposed eyes (**j**), indicative of corneal edema, a hallmark of FECD. **k** Corneal edema in UVA-exposed eyes is accompanied by disruption of cell–cell junctions, as shown by ZO-1 (green) and DAPI (magenta) staining. Scale bar = 20 µm. **l** The UVA-induced FECD mouse model mirrors the clinical upregulation of molecular markers associated with senescence, epithelial–mesenchymal transition (EMT), fibrosis, extracellular matrix (ECM) remodeling, and FECD. Samples: for (**g**–**j**), *n* = 6–8 mice/group; for (**l**), *n* = 3–4 mice/group with technical duplicates per sample. Student’s t-test: *p < 0.05; **p < 0.01; ***p < 0.001; ****p < 0.0001.

The corneal endothelium is a monolayer of hexagonal cells located in the posterior part of the cornea that plays a critical role in maintaining corneal transparency by continuously pumping ions and forming a barrier between the aqueous humor and the corneal stroma [1]. Corneal endothelial (CE) cells are derived from the neural crest, and, like neurons, they are post-mitotic in origin with limited proliferative capacity in vivo [1]. Consequently, when the loss of CE cells exceeds the functional reserve threshold, it leads to corneal edema, necessitating corneal transplantation to restore vision. Throughout the lifetime, CE cells are exposed to cumulative effects of ultraviolet-A light (UVA) which is involved in gradual accumulation of oxidative DNA lesions and progressive decline in the CE cells with age [2, 3]. When antioxidant reserve to combat the continued stress is exhausted due to disease mechanisms, the CE cells exhibit the oxidant-antioxidant imbalance and morphological changes in the monolayer, as seen in Fuchs endothelial corneal dystrophy (FECD) [4, 5]. FECD, which affects approximately 4% of the U.S. population aged over 40 years is an age– and sex– related [4], genetically heterologous disorder with variable expressivity and incomplete penetrance [6]. In FECD, progressive loss of CE cells leads to pleomorphism and polymegethism of the neighboring cells and the deposition of extracellular matrix (ECM) excrescences in the Descemet’s membrane (DM) known as “guttae” that result in visual decline and glare arising from abnormal scattering of light (Fig. 1a–c) [7]. Currently, the only available treatment for FECD is corneal transplantation, which imposes significant socio-economic and medical burdens, and furthermore is complicated by the shortage of human donor corneas available for transplantation [8]. Therefore, the development of pharmacological interventions to bypass the need for corneal transplantation would represent a major advancement in the treatment of FECD.

Studies have shown that post-mitotic differentiated diploid cells under certain conditions re-enter the cell-cycle, but instead of proliferating mitotically they enter the endocycle and polyploidize creating multiple copies of the diploid genome [9, 10]. This process of polyploidy (whole genome duplication) mainly occurs via endocycling in which cells undergo successive genome replication without completing cytokinesis leading to a doubling of the genome with each cycle. Polyploid cells that have three or more copies of the haploid DNA content are thus enlarged and their synthetic capacity is almost equivalent to the multiple cells that would have emerged from cytokinesis [9]. Polyploidy can be a part of a cell’s normal differentiation program, or a response to stress, injury, or pathogenic conditions.

Polyploidy is common in plants and insect cells but also arises in many mammalian cell types, most notably placenta trophoblasts, heart cardiomyocytes, liver hepatocytes, and blood megakaryocytes. Studies to date in these different systems have shed light on how the polyploid cell fate poses an advantage to tissue development and homeostasis. For instance, polyploid cells like sub-perinuclear glia in *Drosophila* nervous system possess large cells necessary to maintain the blood-brain-barrier [11, 12]. Polyploid cells, with multiple copies of the genome, also offer genomic buffering against genotoxic stress [13]. Moreover, in some instances, the DNA damage response pathway is deactivated in polyploid cells preventing apoptosis following genotoxic stress [14–16].

Polyploidy has been found to act as a conserved regulator of stress resistance across the tree of life [17, 18]. Polyploid cells are recognized for their higher growth capacity and play a crucial role in supporting organ growth and maintaining tissue homeostasis, particularly in tissues where mitosis is absent [19]. In cases of tissue repair, several *Drosophila* tissues restore organ size and synthetic capacity solely through polyploidization [20–23]. Polyploidy also contributes to liver [24, 25] and heart regeneration [26], as well as wound healing in mouse kidney tubule epithelial cells [27]. Furthermore, polyploidy–mediated growth may become particularly advantageous over mitosis in restoring tissue tension in response to tissue damage or stress as polyploidization enables cytoskeletal restructuring and upregulation of motor proteins [28, 29]. The absence of mitosis and cell division allows polyploid cells to be more metabolically active, thereby facilitating enhanced macromolecular secretion [28]. Yet, sustained polyploidy has been associated with several pathological conditions [27, 30–37]. In nonalcoholic fatty liver disease, oxidative stress contributes to the development of pathological polyploidy in hepatic cells [38]. Polyploid cardiomyocytes formed after heart failure exhibit dysregulation of mitochondrial biogenesis and defects in electron transport chain complexes [34]. Recently, in chronic kidney disease, polyploidy of tubular kidney cells has been shown to promote senescence and fibrosis [37].

Our previous studies demonstrated that CE cells paradoxically re-enter cell cycle and become arrested in G2/M phase that in turn causes the cells to develop senescent and EMT phenotypes [39, 40]. Although in other organs, the cells that are involved in barrier function respond to cell loss by polyploidization [21, 41], there are no studies investigating whether CE, by re-entering an incomplete cell cycle, undergo endoreplication in response to genotoxic stress such as UVA light. To address this question, we performed a temporal analysis of CE ploidy and morphological changes in response to UVA in the non-genetic mouse model of FECD and correlated the changes with the molecular footprint of FECD markers involved in cell cycle control, senescence, fibrosis, and extracellular matrix deposition seen in guttae [42, 43]. The UVA irradiation of the cornea models CE exposure to chronic oxidative stress as evidenced by findings of oxidative DNA damage and cell cycle arrest in FECD [39, 44]. This approach also enables investigation of modifiable environmental contributors and downstream pathways, including senescence and SASP signaling [43], which are central to our study. While genetic models (e.g., *COL8A2* mutation) have provided valuable insights into early-onset or hereditary forms of FECD, they may not fully capture the cumulative stress-related processes observed in the more common age-related disorder [45]. We detected that UVA led to the initial onset of CE cell endocycling, followed by cellular multinucleation; the latter correlated to the onset of senescent phenotype and production of pro-fibrotic secretory factors. Furthermore, we demonstrated that CE cell polyploidy is mediated by YAP1 signaling, whereas YAP1 inhibition diminishes the secretion of pro-fibrotic factors while rescuing the FECD phenotype in mice. This study provides mechanistic insight into the role of polyploidy in FECD pathogenesis and proposes the prevention of CE cell hyperploidization as a therapeutic strategy for FECD.

## Results

### UVA light induces progressive loss of CE cells and swelling of central cornea, key characteristics of FECD

We used an in vivo UVA-induced, non–genetic mouse model of late-onset FECD [4] to induce oxidative stress in the mouse cornea (Fig. 1d). UVA-induced oxidative DNA damage resulted in polymorphism (Fig. 1e, f) and reduced cell numbers (Fig. 1g, h), recapitulating the CE cell loss observed in FECD patients (Fig. 1b, c). In vivo confocal microscopy using Heidelberg Retinal Tomography (HRT) showed progressive CE cell loss following UVA exposure. Baseline HRT imaging revealed uniformly sized CE cells with the characteristic hexagonal shape, resembling human corneal endothelium. However, UVA exposure disrupted this monolayer, causing progressive CE cell loss, reducing from 2201 ± 31 cells/mm^2^ at baseline to 739 ± 30 cells/mm^2^ on day 14 (Fig. 1g, h). The decreased cell density was concomitant with the development of pleomorphism and polymegathism of CE cells (Fig. 1e, f). The UVA model also replicated corneal edema, a hallmark feature of FECD. This correlated with increased central corneal thickness (CCT) indicative of corneal edema on day 14 (121.4 ± 11.2 μm) compared to baseline (96.9 ± 2.2 μm) as observed by in vivo Anterior Segment Optical Coherence Tomography (AS-OCT) imaging (Fig. 1i, j). Increased corneal edema was associated with disrupted cell junctions, as evidenced by Zonula Occludens-1 (ZO-1) immunostaining of mouse corneas (Fig. 1k).

UVA irradiation also replicated hallmark features of FECD, including cellular senescence, fibrosis, endothelial-mesenchymal transition (EMT), ECM, and clusterin accumulation, as observed in FECD patients [43]. By day 14, our UVA-induced mouse model exhibited significant upregulation of markers associated with senescence: *CDKN2A* (1851-fold) and *CDKN1A* (170-fold); EMT: *ZEB1* (44-fold) and *SNAI1* (164-fold); fibrosis: *FN1* (87-fold) and *ACTA2* (752-fold); ECM: *COL8A2* (69-fold), *COL4A1* (18-fold), *TGFBIp* (53-fold), and FECD: *CLU* (752-fold) compared to control eyes (Fig. 1l), thereby recapitulating the molecular phenomenon observed in FECD patients.

### After UVA-induced oxidative stress, most CE cells either undergo apoptosis or re-enter the cell-cycle

**Fig. 2:**
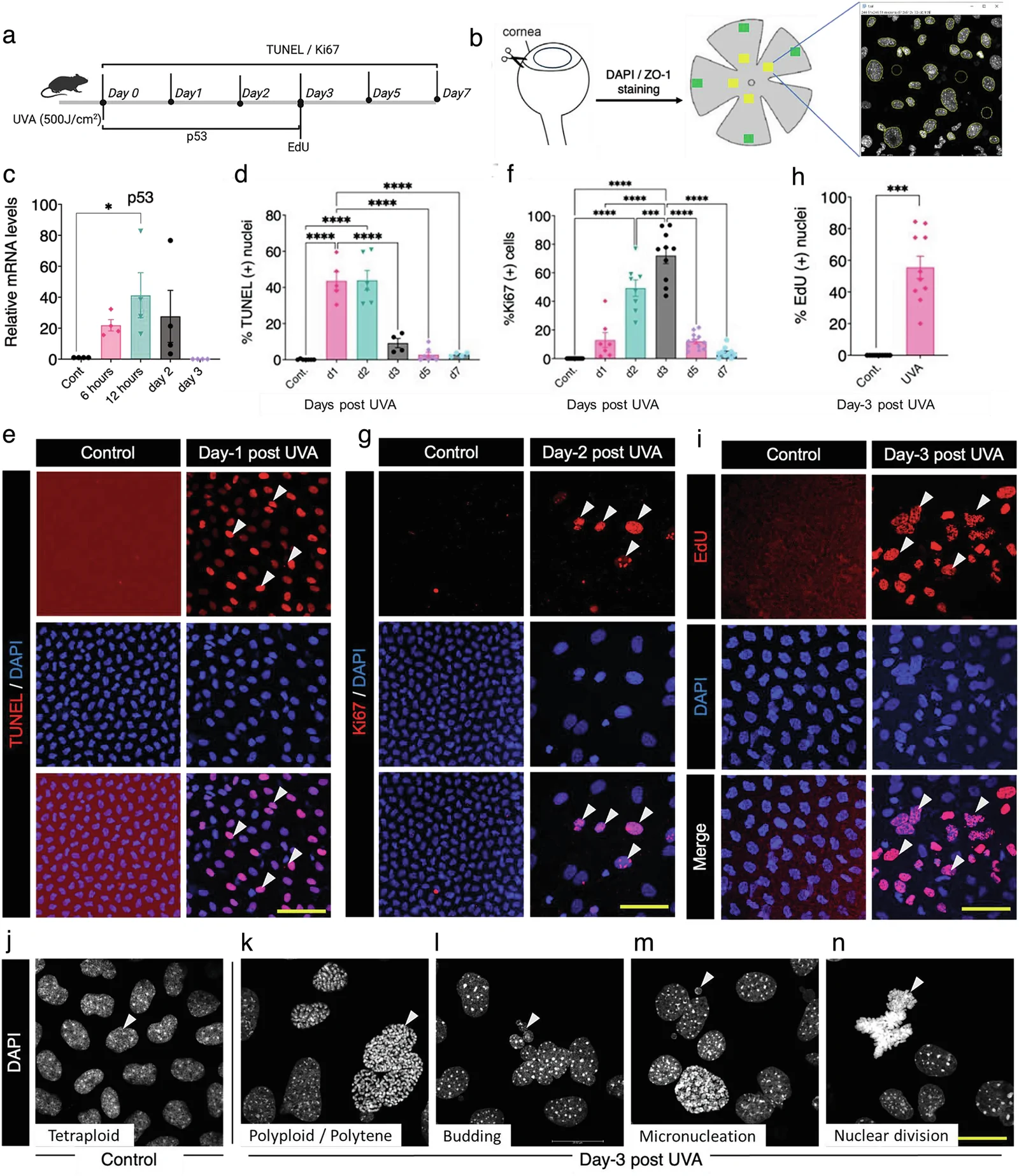
After UVA-induced oxidative stress, most CE cells either undergo apoptosis or re-enter the cell-cycle. **a** Schematic timeline outlining the experimental design to assess UVA-induced apoptosis and cell cycle re-entry in CE cells. **b** Whole-mount mouse corneal cups immunostained with DAPI (nuclear chromatin marker, magenta) for nuclear ploidy analysis. Four central regions of interest were randomly selected per cornea. In the Z-stacked projection of DAPI images individual nuclei were manually outlined and the intensity of DAPI measured with ImageJ was used to calculate the ploidy of individual nuclei. **c** Quantitative RT-PCR shows significant upregulation of p53 mRNA expression 12 h post-UVA exposure. **d**, **e** TUNEL assay demonstrates robust UVA-induced apoptosis in CE cells, peaking at day 1 and day 2 post-exposure (red = TUNEL positive nuclei; blue = DAPI). **f**, **g** Immunostaining for Ki67 indicates cell cycle re-entry of CE cells, with the highest Ki67 positivity observed on day-3 post-UVA (red = Ki67; blue = DAPI). **h**, **i** EdU incorporation assay confirms a significant increase in DNA synthesis at day 3 post-UVA, consistent with cell cycle re-entry (red = EdU positive nuclei; blue = DAPI). **j**–**n** DAPI staining reveals formation of large endopolyploid nuclei or polyteny-like cells at day 3 post-UVA (**k**), which were absent in unexposed control eyes (**j**). Cells that re-entered the cell cycle but failed to complete successful endoreduplication exhibited mitotic abnormalities, including nuclear budding (**l**), micronuclei formation (**m**), and nuclear division (**n**). Scale bars = 20 µm. Samples: for (**c**), *n* = 3–4 mice/group with technical duplicates per sample; for (**d**–**i**), *n* = 3–4 mice/group; 4 images per mouse. Mean ± SEM. One-way ANOVA with Tukey’s HSD test for multiple comparisons. *p < 0.05; **p < 0.01; ***p < 0.001; ****p < 0.0001.

We previously showed that UVA induces oxidative stress in mouse cornea, as indicated by increased levels of reactive oxygen species (ROS) in the aqueous humor [4]. A previous study [46] demonstrated that elevated ROS levels trigger a DNA damage response by activating phosphatidylinositol-3-kinase-related kinases like ATM and ATR, which initiate DNA repair and regulate cell-cycle progression and apoptosis [46, 47]. Using DAPI intensity-based ploidy analysis [48], we analyzed the ploidy of CE nuclei post-UVA (Fig. 2a, b). In our study, UVA exposure significantly upregulated p53 mRNA levels in the corneal endothelium at 12 h, consistent with activation of a DNA damage response (Fig. 2c). While transcriptional upregulation alone does not establish functional activation of p53, we have previously demonstrated that UVA stress leads to increased p53 protein levels and activation of its downstream effector CDKN1A/p21 [4, 39, 40, 43]. Additionally, we have shown activation of phospho-p53 and p53 in FECD human specimens [49]. UVA-induced DNA damage was associated with heightened apoptosis (TUNEL positivity) of CE cells on day-1 (43.6 ± 4.5%) and day-2 (43.9 ± 5.4%) compared to control (0.0 ± 0.4%) (Fig. 2d, e). Our previous in vitro study showed that UVA–induced oxidative DNA damage prompts CE cells to reenter cell-cycle [39]. Therefore, we investigated whether CE cells reenter cell-cycle in vivo after UVA exposure. Immunostaining against Ki67 (a marker for cell cycle reentry) showed that 49.3 ± 5.8% of CE cells reentered cell cycle on day-2, peaking at day-3 (72.0 ± 5.6%) (Fig. 2f, g), suggesting that UVA-induced oxidative DNA damage prompted otherwise non-dividing CE cells to reenter cell-cycle in vivo. However, CE cells are known for their significantly limited proliferative capacity in vivo [1]. These cells are arrested in the G1 phase and typically do not replicate to replace injured or deceased cells [50]. Consequently, we questioned whether CE cells that entered the cell-cycle would successfully complete mitotic cell cycle, giving rise to two independent daughter cells.

We observed that, immediately upon reentering the cell-cycle, CE cells underwent successive rounds of DNA synthesis without undergoing regular karyokinesis, a phenomenon known as endocycling. This incomplete cell-cycle resulted in the formation of large polyploid CE cells. Endocycling of CE cells after UVA exposure was evident through EdU incorporation assay, which showed that 55.5 ± 8.4% of CE cells from UVA-exposed eyes were EdU-positive, compared to controls (0.0 ± 0.0%) (Fig. 2h, i). Furthermore, DAPI staining revealed polyploid nuclei with an abnormally high DNA content in UVA-exposed eyes, compared to the endothelial nuclei in control eyes, which as previously shown [21], were tetraploid and exhibited uniformity in shape, size, and DNA content (Fig. 2j). These polyploid nuclei in UVA-exposed eyes also exhibited abnormally high EdU incorporation, suggesting that they were formed through rapid endocycling post-UVA.

As compared to the naturally occurring tetraploid cells (Fig. 2j), CE cells accumulated additional nuclei following the initial endocycling at day-3, suggesting that the cells may have entered M-phase but failed to undergo nuclear division, a process known as endomitosis thus forming multinucleated, polyploid cells / polytene-like chromosome formation by day-14 (Fig. 2k). At day-3, cells that reentered the cell cycle but failed to undergo successful endocycling exhibited mitotic defects such as nuclear budding (Fig. 2l) [51], micronuclei formation (Fig. 2m) [52, 53], or nuclear division (Fig. 2n) [54].

### CE cells endocycle and then endomitose after reentering cell-cycle

**Fig. 3:**
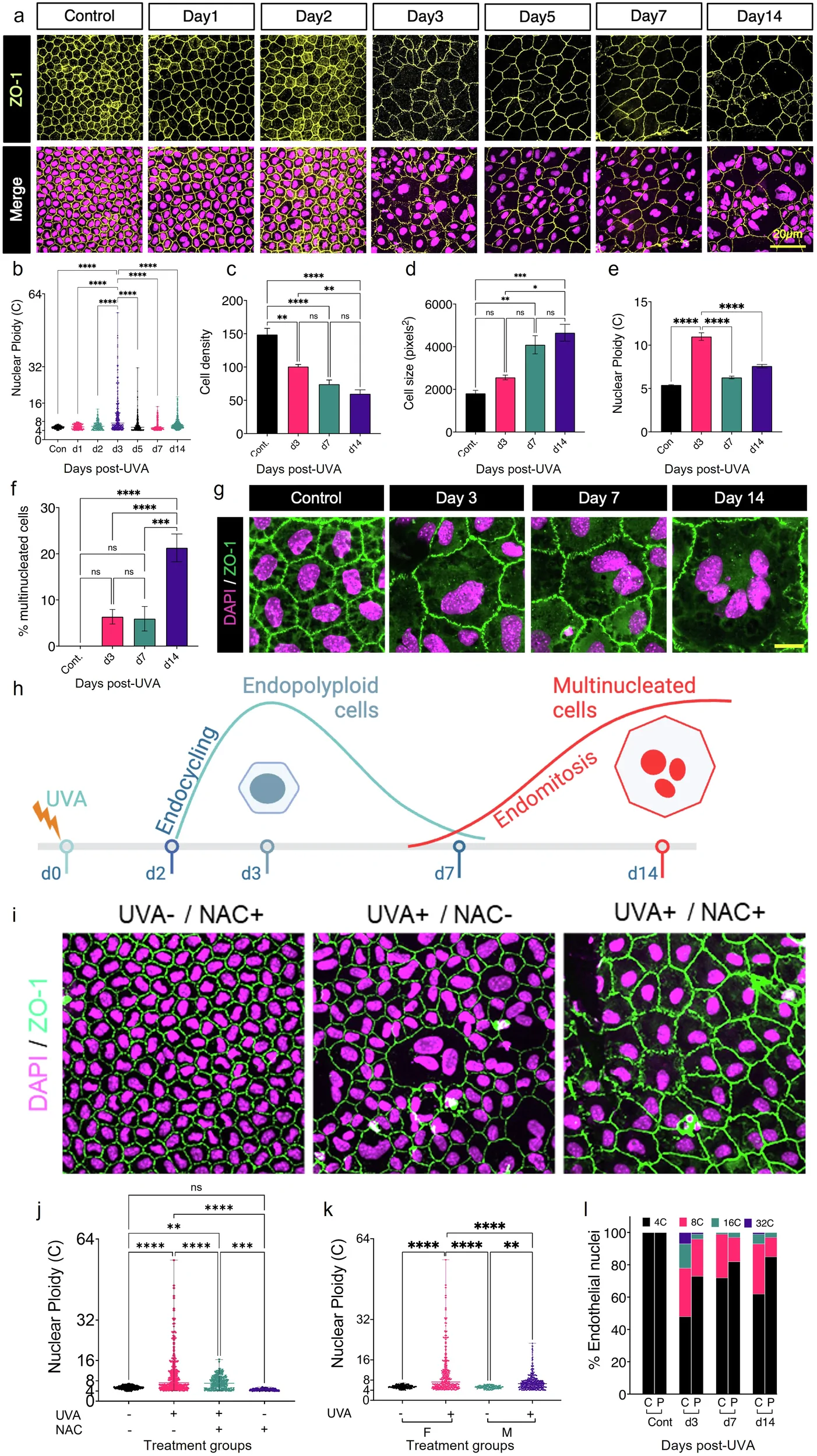
CE cells endocycle and then endomitose after reentering cell-cycle. Entry into endocycle post-UVA is dependent on ROS, animal sex and topological location within cornea. **a**, **b** DAPI staining (magenta) showing a time-dependent increase in nuclear ploidy following UVA exposure (**a**). Scatter plots (median ± range) of nuclear ploidy per nucleus (1 dot = 1 nucleus), showing a peak in polyploidization at day 3 post-UVA (**b**). Some nuclei exhibit extreme polyploidy at this time point. **c**, **d** ZO-1-based CE cell counts demonstrate progressive cell loss post-UVA exposure (**c**). Surviving CE cells show a compensatory increase in cell size, inversely correlated with decreasing cell density (**d**). **e** Nuclear ploidy peaks at day 3 post-UVA but subsequently decreases with the emergence of multinucleated cells. **f**, **g** A significant increase in multinucleated, polyploid CE cells is observed on day 14 post-UVA. Scale bars = 20 µm. **h** Schematic summary of the sequential cellular events following UVA exposure: surviving CE cells re-enter the cell cycle by day 2, undergo endocycling to form endopolyploid nuclei by day 3, and subsequently transition to endomitosis, forming multinucleated polyploid cells peaking at day 14 post-UVA. **i**, **j** Pretreatment with the antioxidant N-acetylcysteine (NAC) significantly attenuated UVA-induced polyploidization. **k** Polyploidization was more pronounced in female mice compared to males. **l** Quantification of polyploid CE cells in the central versus peripheral cornea shows greater polyploidization centrally. Nuclear ploidy values were categorized into the following bins: 4 C (3.0–5.9 C), 8 C (6.0–12.9 C), 16 C (13.0–24.9 C), and 32 C ( > 25.0 C). Samples: *n* = 3, 4 mice/group; 4 images per mouse. Mean ± SEM. One-way ANOVA with Tukey’s HSD test for multiple comparisons. *p < 0.05; **p < 0.01; ***p < 0.001; ****p < 0.0001.

Our ploidy measurements of CE nuclei obtained at different time points post-UVA showed that CE cells from unexposed eyes are at least twice as large and contain twice the DNA content to that of the epithelial nuclei, indicating that they are tetraploid, as previously reported [21], with an average ploidy of 5.4 ± 0.0 C. However, after UVA exposure, nuclear ploidy increased on day-1 (5.7 ± 0.1 C) and day-2 (6.3 ± 0.1 C), reaching maximum values on day-3 (11.0 ± 0.4 C) (Fig. 3a, b), with some nuclei exhibiting extreme polyploidy, reaching up to 64 C. Following the attainment of maximum ploidy values on day-3, CE cells appeared to endomitose resulting in reduced nuclear ploidy. Nuclear ploidy values dropped to 6.4 ± 0.2 C, 6.3 ± 0.2 C and 7.2 ± 0.1 C on day-5, day-7, and day-14 respectively. The results suggest that CE nuclei endocycle immediately after UVA-induced oxidative DNA damage and then reduce nuclear ploidy by endomitosis (Fig. 3b).

We further investigated the kinetics of changes in cell size and multinucleation at various time points following UVA exposure. ZO-1 immunostaining revealed a progressive decline in cell density following UVA exposure. Cell densities (cells/field) on day-3 (100.0 ± 3.3), day-7 (74.1 ± 6.4), and day-14 (59.8 ± 5.9) were significantly lower than those of control eyes (148.7 ± 9.4) (Fig. 3c). The decline in cell density, as observed by HRT imaging (Fig. 1g, h) was accompanied by cellular pleomorphism, polymegethism and an overall increase in average size of the surviving cells, as evident from ZO-1 immunostaining (Fig. 3a). Average cell sizes (pixels^2^) significantly increased on day-7 (4088.4 ± 425.3), and day-14 (4652.7 ± 394.9), compared to the control group (1808.0 ± 145.5) (Fig. 3d). This inverse relationship between cell density and cell size suggests that, following UVA exposure, surviving CE cells undergo cellular hypertrophy in an attempt to restore tissue homogeneity and CE functions, as observed clinically [55].

With the onset of active endocycling, nuclear ploidy significantly increased on day-3 (11.0 ± 0.4 C) compared to the control (5.4 ± 0.0 C) but decreased to 6.3 ± 0.2 C and 7.2 ± 0.1 C on day-7 and day-14 respectively (Fig. 3e). This reduction in nuclear ploidy was associated with a progressive increase in the percentage of multinucleated cells on day-3 (5.4 ± 1.4%), day-7 (6.8 ± 3.0%), and day-14 (21.3 ± 3.0%) (Fig. 3f) post-UVA, which were completely absent in control eyes. These findings suggest that UVA prompts active endocycling in mouse CE cells until day-3, followed by endomitosis that results in multinucleated cells (Fig. 3g, h).

### Entry into endocycle post-UVA is dependent on ROS, animal sex and topological location within cornea

Previous studies have shown that N-acetylcysteine (NAC), due to its antioxidant and reactive oxygen species (ROS)-scavenging properties, exerts cytoprotective effects in mouse models of early-onset FECD [4]. Therefore, we investigated whether quenching UVA-induced oxidative stress could reduce polyploidization of CE cells. We administered NAC (1000 mg/kg, i.p.) to 8-week-old female C57BL/6J mice 1 h prior to UVA exposure and assessed changes in nuclear ploidy on day-3. UVA-exposed CE cells exhibited multinucleated cells (Fig. 3i) and increased ploidy, which was reduced by NAC pre-treatment (7.3 ± 0.2 C) (Fig. 3j), suggesting that CE cell polyploidization is a response to oxidative stress.

Since FECD is a female-predominant disorder [56], we investigated how sex influences polyploidy changes after UVA. We exposed 8-weeks-old female and male mice to UVA and analyzed nuclear ploidy on day-3 following exposure. CE cells from UVA-exposed female mice exhibited robust polyploidization (nuclear ploidy = 11.0 ± 0.4 C) compared to the unexposed female mice (5.4 ± 0.0 C) (Fig. 3k). In contrast, CE cells from UVA-exposed male mice showed weaker polyploidization after UVA (7.2 ± 0.1 C) compared to unexposed male mice (5.2 ± 0.0 C). Nuclear ploidy in the UVA-exposed female mice was significantly higher than in UVA-exposed male mice, indicating that females undergo more pronounced polyploidization in response to UVA. The higher incidence of FECD in women [56] and greater CE cell loss previously reported in female mice [57] following UVA may be attributed to a greater propensity of female CE cells to polyploidize after oxidative stress.

Studies have shown that UVA light transmission is higher in the central cornea compared to the periphery [3], and CE cells in the central region experience greater levels of oxidative DNA damage [39]. To better evaluate topographical differences in CE cell polyploidization, we compared ploidy changes between the central and peripheral regions of the mouse cornea in 2-month-old female C57BL/6 mice following UVA exposure. At day-3, the nuclear ploidy in the central cornea (11.0 ± 0.4 C) was significantly higher than that in the periphery (6.7 ± 0.2 C). The proportion of polyploid cells was consistently higher in the central cornea than in the periphery at all other evaluated time points (Fig. 3l).

### Multinucleated CE cells are more pro-senescent and pro-fibrotic than mononucleated cells

**Fig. 4:**
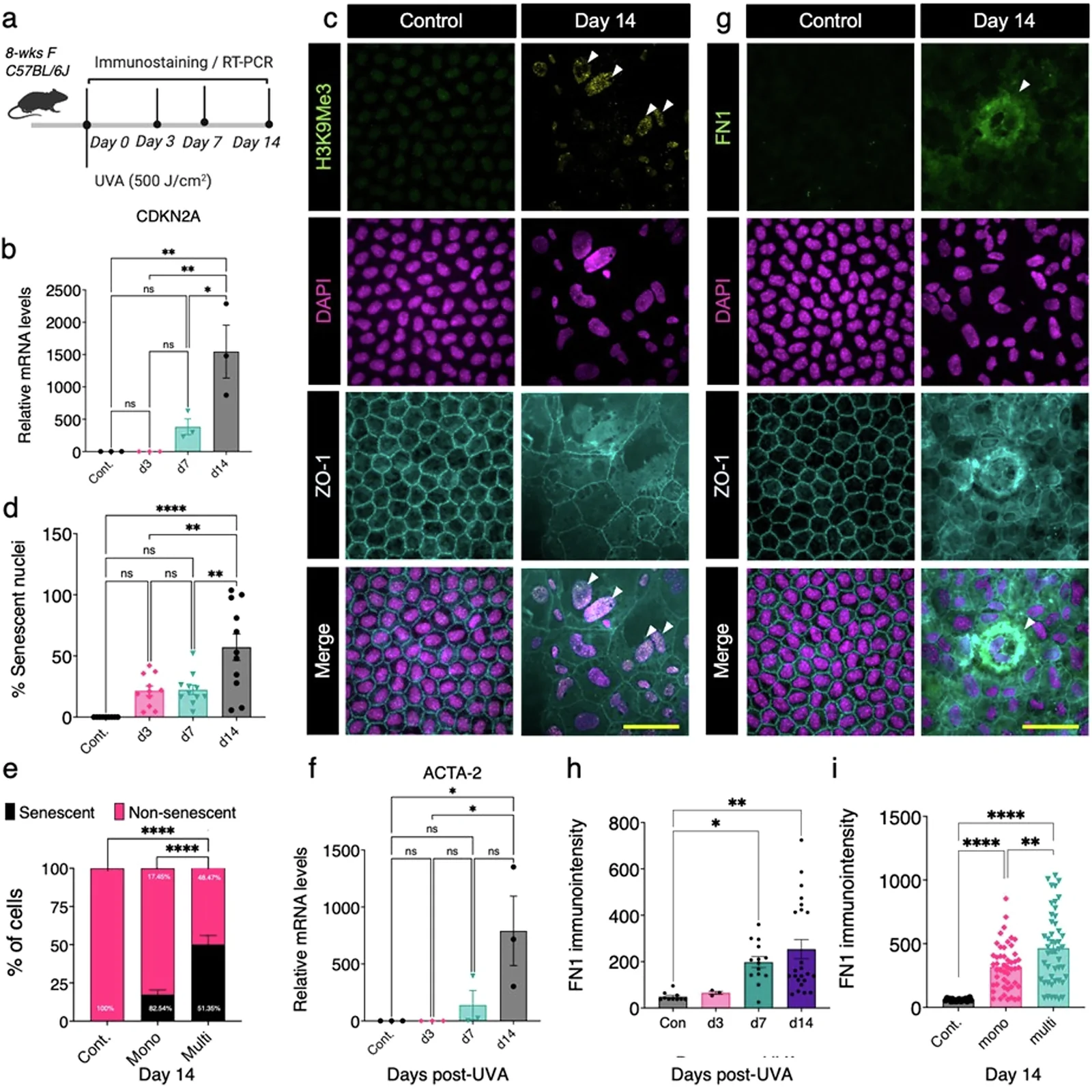
Multinucleated CE cells are pro-senescent and pro-fibrotic than mononucleated cells. **a** Experimental timeline to assess the temporal dynamics of senescence and fibrosis in mouse CE following UVA exposure. Eight-week-old female C57BL/6J mice were exposed to UVA light (500J/cm²); corneas were harvested at 3-, 7-, and 14-days post-UVA for immunohistochemistry (IHC) or RT-PCR analyses. **b** RT-PCR analysis showed a time-dependent increase in *CDKN2A* mRNA expression (a marker of senescence), peaking at day 14 post-UVA. **c**, **d** Immunostaining against H3K9me3 (green), a senescence marker, demonstrated a progressive increase in the percentage of senescent CE cells over time, with a maximum at day 14. **e** At day-14 post-UVA, the percentage of multinucleated polyploid cells which were senescent was higher than the percentage of mononucleated cells which were senescent. **f** RT-PCR analysis showed progressive increase in mRNA expression level of *ACTA2* (a fibrosis marker), also peaking at day-14 post-UVA. **g**, **h** Immunostaining against fibronectin-1 (FN1, green) showed increasing extracellular FN1 deposition over time, with maximal accumulation at day 14 post-UVA. **i** At day 14, FN1 intensity was significantly higher in multinucleated CE cells compared to mononucleated cells. Each dot represents FN1 signal intensity per individual cell. Samples: *n* = 3 mice/group; 2 technical duplicates (for RT-PCR) or 4 images (for IHC) per mouse; Scale bars = 20 μm. Mean ± SEM. One-way ANOVA followed by Tukey’s HSD post-hoc test. *p < 0.05; **p < 0.01; ***p < 0.001; ****p < 0.0001.

The emergence of multinucleated polyploid cells was accompanied by an increase in cellular senescence and fibrosis. RT-PCR analysis revealed that CE cells began expressing the senescence marker p16 (*CDKN2A*) after day-7, with expression peaking at day-14 (1546-fold) (Fig. 4a, b). This finding was corroborated by immunohistochemistry using H3K9me3, a marker of senescent nuclei (Fig. 4c). While CE cells in the control group lacked senescent cells (0.0 ± 0.0%), there was a steady increase in the percentage of senescent cells at day-3 (21.5 ± 3.7%), day-7 (22.4 ± 3.9%) and day-14 (57.2 ± 10.49%) (Fig. 4d). Further, we analyzed cellular senescence in relation to the polyploidy status of CE cells. At day-14, the time point at which senescence peaked, only 17.5 ± 0.2.9% of mononucleated cells were H3K9me3 positive, whereas a significantly larger proportion of multinucleated cells were H3K9me3 positive (50.1 ± 0.5%) (Fig. 4e), suggesting that multinucleated CE cells are pro-senescent compared to mononucleated polyploid cells.

Furthermore, RT-PCR analysis showed that CE cells began expressing *ACTA2*, a marker of fibrosis, after day-7, with expression reaching significantly elevated levels by day-14 (790-fold) (Fig. 4f). Immunostaining against fibronectin, another fibrosis marker, also revealed a progressive increase in FN-1 deposition (Fig. 4g), starting at 48.7 ± 5.9 (mean gray value) in controls and escalating to 65.4 ± 6.6, 197.8 ± 50.9, and 253.85 ± 41.14 at day-3, day-7, and day-14, respectively (Fig. 4h). Notably, at day-14, FN-1 deposition in multinucleated cells (465.0 ± 41.0) was significantly higher than in mononucleated cells (318.31 ± 25.80) (Fig. 4i).

Overall, the results demonstrate a progressive increase in senescence and fibrosis following UVA exposure, with both peaking at day-14. Beyond this point, substantial cell loss hindered reliable quantification. Consequently, all subsequent analyses were restricted to the first 14 days to accurately capture early cellular responses to UVA-induced stress. These changes began to emerge after day-7, coinciding with the cessation of active endocycling and onset of endomitosis, which led to the formation of multinucleated polyploid cells. Levels of senescence and fibrosis were significantly higher in multinucleated cells than in mononucleated cells. These findings provide initial evidence that multinucleated cells are more pro-senescent and pro-fibrotic compared to mononucleated cells and may play a greater role in the progression of FECD.

### Endocycling of CE cells is mediated by YAP1 signaling

**Fig. 5:**
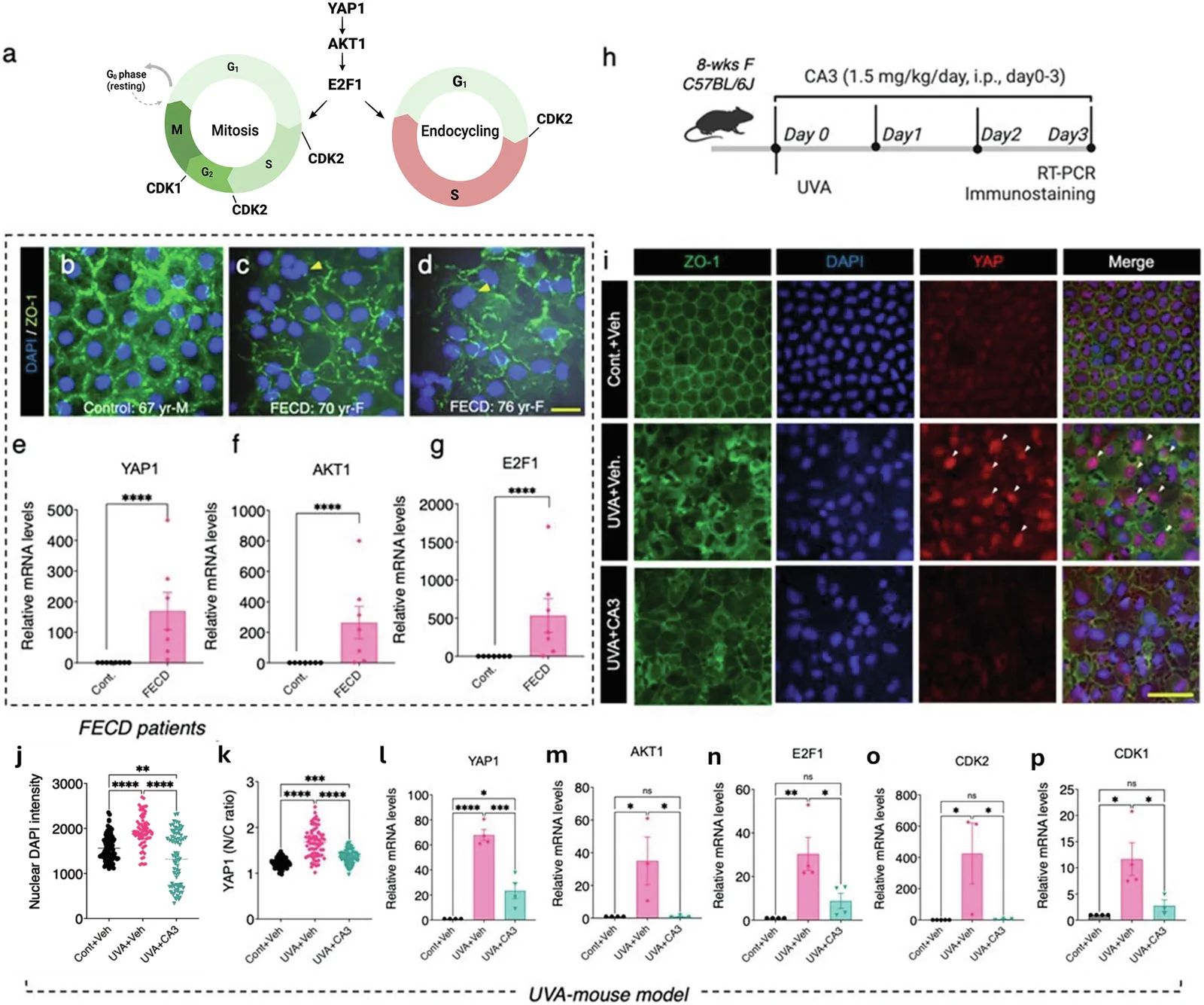
Endocycling of CE cells is mediated by YAP1 signaling. **a** Schematic model summarizing the key regulatory proteins governing the mitotic cell cycle versus the endocycle. **b**–**d** DAPI/ZO-1 staining of human corneal endothelium showed normal morphology of CE cells in healthy donors (**b**) and presence of polyploid cells (yellow arrowheads) in FECD patients (**c**, **d**). Scale bars = 20 μm. **e**–**g** RT-PCR analysis of human CE tissue revealed significantly increased mRNA expression of *YAP1*, *AKT1*, and *E2F1* in FECD patients. **h** Experimental timeline: 8-week-old female C57BL/6J mice were exposed to UVA (500 J/cm^2^). Mice were administered YAP1 inhibitor CA3 (1.5 mg/kg, i.p.) 1 h prior to UVA exposure and on days 1, 2, and 3 post-UVA. Corneas were harvested on day-3 for immunostaining or RT-PCR analysis. UVA exposure induced nuclear translocation of YAP1 (**i**), as evidenced by an increased nuclear DAPI intensity (**j**), nuclear-to-cytoplasmic (N/C) ratio of YAP1 immunointensity (**k**). CA3 treatment significantly reduced DAPI intensity and nuclear translocation of YAP1. Scale bar = 20 μm. (*n* = 3mice; 90 cells per group). RT-PCR analysis demonstrated that *Yap1* (**l**), *Akt1* (**m**), and *E2f1* (**n**) mRNA levels were upregulated following UVA exposure. This upregulation was significantly attenuated by CA3 treatment. UVA also induced the expression of S-phase cyclin-dependent kinases *CDK2* (**o**) and *CDK1* (**p**), consistent with activation of the endocycle, and these effects were reversed by CA3. Samples: for (**i**), *n* = 3 mice/group, 4 images/mouse; for (**l**–**p**), *n* = 3 mice/group with biological duplicates per sample. Mean ± SEM, one-way ANOVA with Tukey’s HSD. For (**b**–**g**) (*n* = 8 FECD specimens and *n* = 6 healthy donors), technical duplicates per sample. Unpaired Student’s t-test: *p < 0.05; **p < 0.01; ***p < 0.001; ****p < 0.0001. See Supplementary Table 2 for cornea donor information and details on tissue preservation details.

Previous studies have shown that Yes-associated protein 1 (YAP1), and its *Drosophila* ortholog Yorkie, regulates endoreplication by modulating G/S phase genes (Fig. 5a) [21, 37, 58]. We investigated whether CE cell polyploidy and YAP1 signaling are involved in the pathogenesis of FECD in human patients. DAPI/ZO-1 staining revealed the presence of multinucleated cells in corneal endothelium of FECD patients, which were absent in healthy donor samples (Fig. 5b–d). This observation was consistent with RT-PCR analysis, which showed significant upregulation of *YAP1* (190-fold; Fig. 5e), *AKT1* (365-fold; Fig. 5f), and *E2F1* (461-fold; Fig. 5g) in the corneal endothelium of FECD patients compared to healthy cadaveric donors.

To assess YAP1’s role in CE cell endocycling in the UVA-FECD mouse model, 8-week-old female mice were treated with the selective YAP1 transcriptional activity inhibitor CA3 (1.5 mg/kg, i.p.) 1 h prior to UVA exposure and on days 1–3 post-UVA. Corneal endothelium was collected 1 h after the final injection on day-3 (Fig. 5h). By day-3 post-UVA, significant nuclear translocation of YAP1 was observed, which was markedly reduced in CA3-treated mice. The nuclear DAPI intensity values increased from 1560 ± 288 (control) to 1943 ± 355 (UVA+vehicle), which significantly reduced to 1319 ± 592 when treated with CA3 (Fig. 5i, j). Additionally, nuclear-to-cytoplasmic (N/C) ratio of YAP1 intensity was significantly elevated in the UVA+vehicle group (1.66 ± 0.04) compared to controls (1.31 ± 0.01), and this elevation was significantly attenuated in the UVA + CA3 group (1.35 ± 0.02) (Fig. 5k). RT-PCR analysis revealed a significant upregulation of *YAP1* mRNA levels following UVA exposure (67-fold), which was significantly reduced with CA3 treatment (23-fold). YAP1 activation was associated with the upregulation of its downstream effectors AKT Serine/Threonine Kinase 1 (AKT1) and E2F transcription factor 1 (E2F1), which previously were implicated in promoting polyploidy [37]. E2F1, which drives G1-S transition and DNA synthesis, was specifically elevated after UVA, whereas E2F7 and E2F8, known repressors of endoreplication, were not (Supplementary Fig. 1). This selective E2F modulation may facilitate polyploidization despite the presence of E2F1, as shown in other cell types [59]. *AKT1* mRNA levels were significantly upregulated after UVA exposure (35-fold) and were substantially reduced following CA3 treatment (1-fold). Similarly, *E2F1* mRNA expression was significantly increased after UVA exposure (30-fold) and was attenuated with CA3 treatment (9-fold). Upregulation of YAP1 signaling was also associated with upregulation of cyclin-dependent-kinases *CDK2* (425-fold) and *CDK1* (12-fold), both of which were significantly downregulated following CA3 treatment (*CDK2* 5-fold; *CDK1* 3-fold) (Fig. 5l–p). The results suggest that UVA-induced activation of YAP1 promotes upregulation of its downstream effectors AKT1 and E2F1, ultimately leading to increased expression of S and G2/M phase CDKs.

### YAP1 inhibition ameliorates senescence and fibrosis in UVA-mouse model

**Fig. 6:**
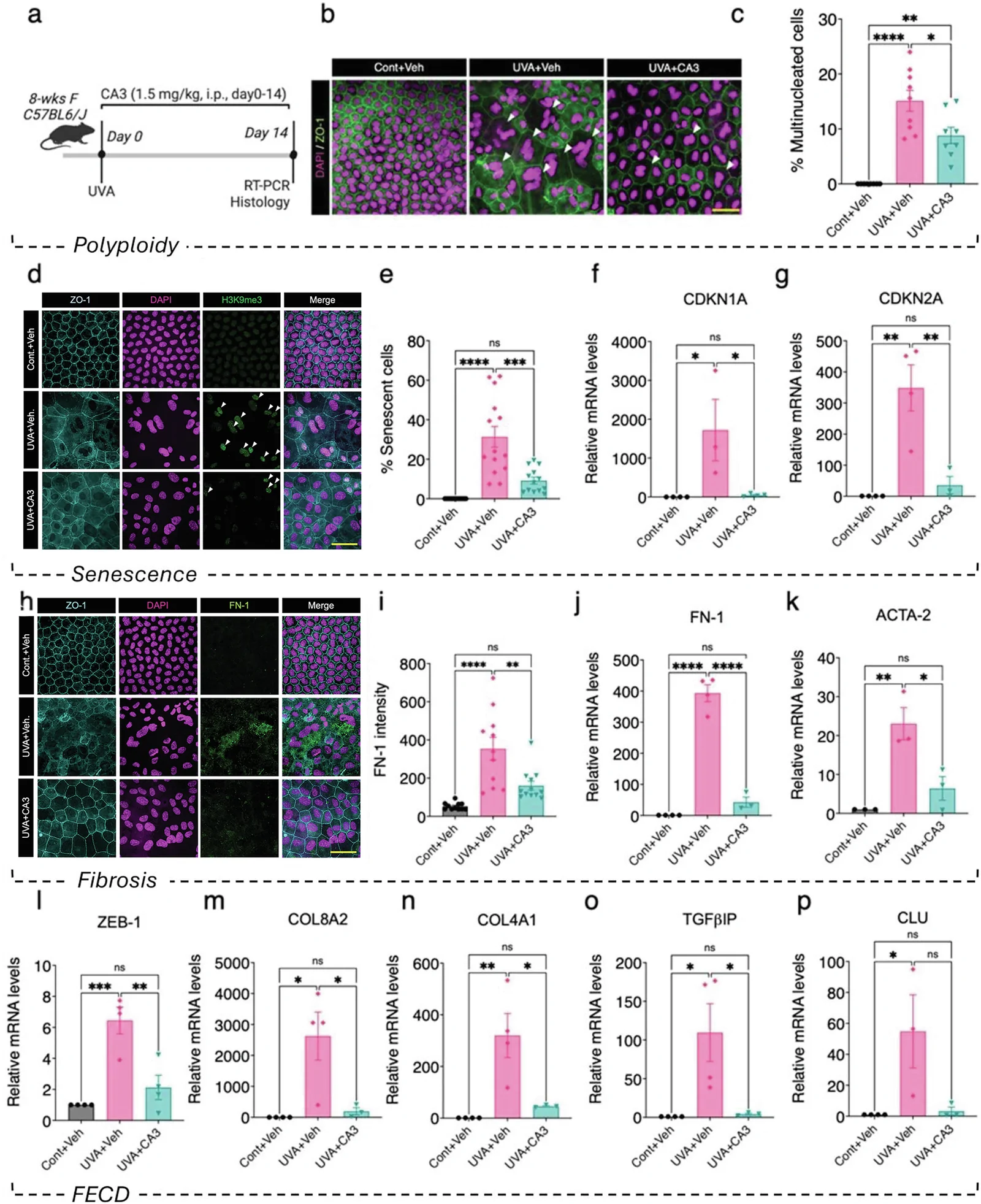
YAP1 inhibition ameliorates senescence and fibrosis in FECD mouse model. **a** Schematics of the CA3 treatment regime. Eight-week-old female C57BL/6J mice were exposed to UVA (500 J/cm^2^). Mice received daily injections of either CA3 (1.5 mg/kg, i.p.) or vehicle from day 0 to day 14. Corneas were harvested on day 14 for immunostaining and RT-PCR. **b**, **c** DAPI/ZO-1 staining revealed a significant increase in multinucleated CE cells on day 14 post-UVA exposure. CA3 treatment markedly reduced the percentage of multinucleated cells. **d**, **e** Immunostaining for H3K9me3 (senescence marker) showed a significant increase in the percentage of senescent cells in the UVA + vehicle group, which was significantly reduced in the UVA + CA3 group. **f**, **g** RT-PCR analysis demonstrated upregulation of senescence-associated genes *CDKN1A* (**f**) and *CDKN2A* (**g**) following UVA exposure; both were significantly downregulated upon CA3 treatment. **h**, **i** Immunostaining against fibronectin-1 (FN1) revealed elevated extracellular matrix deposition in the UVA+vehicle group, which was reduced by CA3 administration. RT-PCR analysis showed increased expression of fibrosis markers *FN1* (**j**) and *ACTA2* (**k**) in the UVA+vehicle group, both of which were significantly downregulated by CA3. RT-qPCR analysis further demonstrated upregulation of other FECD markers *ZEB1* (**l**), *COL8A2* (**m**), *COL4A1* (**n**), and *TGFβIP* (**o**) and *CLU* (**p**) in the UVA-treated group. These markers were significantly downregulated with CA3 treatment. Samples: *n* = 3 mice per group; 4 images or technical duplicates per sample. Mean ± SEM. Scale bars = 20 μm. One-way ANOVA with Tukey’s HSD post-hoc test. *p < 0.05; **p < 0.01; ***p < 0.001; ****p < 0.0001.

We speculated that inhibiting YAP1 could prevent CE cell polyploidization, thereby reducing cellular senescence and fibrosis. To test this, we administered CA3 (1.5 mg/kg/day, i.p.) to 8-week-old female C57BL/6J mice 1 h prior to UVA exposure daily from day-1 to day-14 post-UVA and assessed its effects on FECD progression (Fig. 6a). At day-14, CA3 treatment significantly reduced the percentage of multinucleated cells (8.80 ± 1.4%) compared to UVA+vehicle group (15.1 ± 1.90%) (Fig. 6b, c). This reduction in multinucleation correlated with a decrease in cellular senescence. At day-14, UVA+vehicle group exhibited a significantly higher proportion of H3K9me3-positive senescent cells (32.2 ± 5.4%) compared to controls (0.00 ± 0.00%), which was significantly reduced to 9.20 ± 1.7% with CA3 treatment (Fig. 6d, e). These findings were corroborated by RT-PCR, which showed significant upregulation of senescence markers (*CDKN1A* 955-fold and *CDKN2A* 314-fold) following UVA exposure, and a reduction by 20-fold (*CDKN1A;* *p* < 0.05) and 7-fold (*CDKN2A;* *p* < 0.01) with CA3 treatment (Fig. 6f, g).

Additionally, YAP1 inhibition reduced FN-1 deposition in CE cells after UVA exposure. At day-14, FN-1 intensity in the UVA+vehicle group (341.5 ± 58.9 arbitrary units) was significantly elevated compared to the control+vehicle group (52.5 ± 5.7). CA3 treatment reduced FN-1 deposition to 160.6 ± 23.5 (Fig. 6h, i). UVA significantly upregulated fibrosis markers *FN-1* (394-fold) and *ACTA2* (25-fold) compared to non-UVA controls. CA3 treatment reduced the levels of *FN-1* by 8-fold (*p* < 0.0001) and *ACTA2* by 4-fold (*p* < 0.05) (Fig. 6j, k).

Beyond mitigating senescence and fibrosis, YAP1 inhibition also attenuated key FECD markers. Following UVA, RT-PCR analysis showed significant upregulation of *ZEB1* (7-fold), an EMT marker observed in FECD, which was reduced by 305-fold (*p* < 0.01) after CA3 treatment (Fig. 6l). Similarly, UVA upregulated hallmark ECM markers that are observed in FECD, such as *COL8A2* (2169-fold), *COL4A1* (248-fold), and *TGFBIp* (133-fold). CA3 treatment substantially reduced the expression of these markers by 10-fold (*COL8A2*, *p* < 0.05); 6-fold (*COL4A1*, *p* < 0.05); and 4-fold (*TGFBIP*, *p* < 0.05) (Fig. 6m–o). Additionally, *CLU* (54-fold), which was significantly upregulated following UVA exposure, was reduced by 11-fold after CA3 treatment (Fig. 6p). These results confirm that, in addition to suppressing senescence and fibrosis, YAP1 inhibition also ameliorates other molecular markers of FECD.

### YAP1 inhibition rescues FECD pathogenesis in a UVA-induced mouse model

**Fig. 7:**
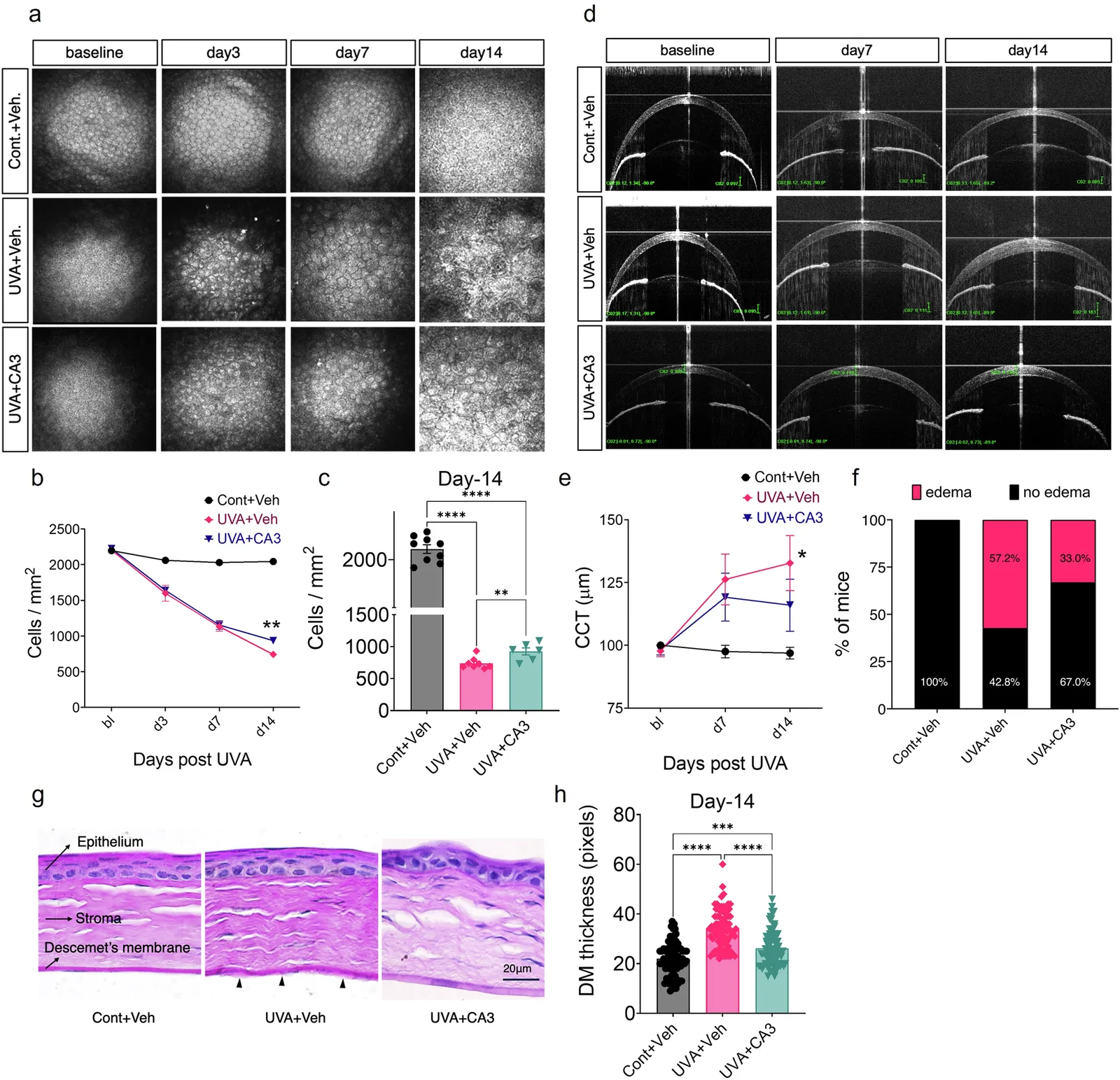
YAP1 inhibition rescues FECD phenotype in UVA-mouse model. Eight-week-old female C57BL/6J mice were exposed to UVA (500 J/cm^2^). Mice received daily injections of either CA3 (1.5 mg/kg, i.p.) or vehicle from day 0 to day 14. **a** In vivo imaging by Heidelberg Retinal Tomography (HRT) and anterior segment optical coherence tomography (AS-OCT) was performed at baseline (1 day prior to UVA exposure), and on days 3, 7, and 14 post-UVA. **b**, **c** HRT imaging revealed a significant rescue in CE cell density in CA3-treated mice at day 14 post-UVA compared to the vehicle group. **d**–**f** AS-OCT imaging demonstrated significant rescue of corneal edema in the CA3-treated group (d-e). At day 14 post-UVA, 57.2% of mice in the UVA + vehicle group exhibited corneal edema (defined as central corneal thickness, CCT > 120 µm), whereas only 33% of mice in the UVA + CA3 group developed edema (*n* = 10 mice per group) (**f**). **g**, **h** PAS staining showed a significant increase in Descemet’s membrane thickness following UVA exposure, which was markedly reduced with CA3 treatment. Scale bar = 20 μm. Sample: for (**b**, **c**), *n* = 6–8 mice per group; (**g**, **h**), *n* = 3 mice per group; 3 sections per eye, 3 images per section. Mean ± SEM, one-Way ANOVA, all groups compared against all groups, Tukey’s HSD. *p < 0.05, **p < 0.01, ***p < 0.001, ****p < 0.0001.

YAP1 inhibition (CA3 1.5 mg/kg, days 1–14) resulted in a significant rescue of CE cell loss following UVA exposure. Consistent with previous findings [4], the UVA+vehicle group exhibited substantial CE cell loss, declining from 2183 ± 18 cells/mm^2^ at baseline to 1598 ± 112 cells/mm^2^ at day-3 and 1133 ± 63 cells/mm^2^ at day-7. However, a significant change was observed at day 14 between UVA+Vehicle (742±22cells/mm^2^) and UVA + CA3 group (935 ± 39 cells/mm^2^) (Fig. 7a, b). By day-14 CE cell density in UVA + CA3 group was significantly higher than in the UVA+vehicle group (Fig. 7c). Mice treated with CA3 showed a 19.25% rescue in CE cell density compared to the UVA+vehicle group.

Furthermore, YAP1 inhibition significantly ameliorated corneal edema at day-14 post UVA exposure. At day 14, the UVA+vehicle group showed a marked increase in (CCT: 126.6 ± 10.6 μm) compared to the control+vehicle group (96.9 ± 2.4 μm), whereas the UVA + CA3 group exhibited significantly reduced edema (CCT: 116.0 ± 10.4 μm) (Fig. 7d, e). Notably, on day-14 post-UVA, 57.2% of mice in the UVA+vehicle group exhibited corneal edema (CCT>120 μm), whereas only 33.0% of mice in the UVA + CA3 group showed similar signs of edema (Fig. 7f). Additionally, YAP1 inhibition significantly reduced thickening of the DM, a hallmark feature commonly observed in FECD patients [60]. Periodic Acid-Schiff (PAS) staining revealed a significant increase in DM thickness following UVA exposure (33.8 ± 1.5 pixels) compared to controls (22.2 ± 1.4 pixels). CA3 treatment significantly reduced DM thickness to 26.1 ± 1.4pixels (Fig. 7g, h). Thus, by preventing polyploidy of CE cells by YAP1 inhibition we observed a significant decrease in senescence, fibrosis, EMT, ECM and FECD markers in association with a significant rescue of CE cell loss, corneal edema, and DM thickening in this FECD mouse model.

## Discussion

The remarkable similarity between adult Drosophila abdominal epithelium and CE, both monolayers that serve as barrier function, led to the study of polyploidy in response to UVA-induced injury in the cornea. Both cell types are quiescent and do not exhibit active cycling, although they have a potential to reenter the cell cycle in response to injury [39, 40]. In Drosophila, quiescent epithelial cells respond to puncture by reentering the cell cycle and growing via polyploidy, or become multinucleated albeit by cell fusion to restore tissue integrity [20]. Similarly, CE cells, which are made up of a single sheet of diploid cells that lie in direct contact with aqueous humor and control transportation of water ions in response to osmotic pressure, are exposed to surgical and degenerative insults. They are in the direct path of UVA light exposure, which has been shown to be an environmental risk factor for FECD development [61]. In this study, we demonstrate for the first time, that in response to UVA injury, CE reenter the cell cycle and initiate polyploidization as seen in FECD human specimens. Furthermore, the evaluation of the biological relevance and consequences of CE polyploidization demonstrates that initial polyploidization serves as a function-preserving stress response, but with persistent stress, as seen during chronic degenerative process, there is activation of the pro- fibrotic pathway, resulting in loss of corneal function [42, 43, 62].

Previously, we have demonstrated that UVA irradiation of cornea generates ROS in aqueous humor, as seen in human FECD aqueous specimens [4, 57], that by inducing macromolecular damage, leads to CE cell apoptosis and cell loss [4, 49] thus manifesting in a FECD-like phenotype in vivo [4]. Here we show that after UVA exposure, a portion of CE cells undergo apoptosis (TUNEL positivity), resulting in an immediate decline in CE cell density. Interestingly, by day-2, surviving CE cells that sustained sublethal DNA damage reentered the cell cycle (Ki67 positivity) and activated DNA synthesis (EdU positivity), that are consistent with activation of polyploidy, aimed at restoring the CE cell density and function.

Notably, after reentering the cell cycle, CE cells underwent endocycling, engaging in multiple rounds of DNA synthesis without nuclear division, thus generating giant polyploid nuclei. Specifically, at day-3 following UVA, cells that reentered the cell cycle but failed to undergo successful endocycling exhibited mitotic defects such as (a) nuclear budding, which is a conserved, alternative pathway for nucleo-cytoplasmic communication, broadly observed across species as it is mainly associated with cellular stress responses and depolyploidization [51]; (b) micronuclei formation, where small nuclei formed from chromosomes or its fragments that are excluded during cell division. They indicate genotoxic stress and chromosomal instability, serving as useful markers for DNA damage, mitotic errors, and cellular stress responses [52, 53]; and (c) polytene-like chromosome formation, where oversized, banded structures form by repeated DNA replication without division. In polyploid cells, similar polytene-like chromosomes can form, showing banding patterns but differing slightly in organization, reflecting specialized adaptations of polyploidy and endocycle variations [54]. These structures serve as validated indicators of genotoxic stress and DNA damage of CE cells following UVA exposure.

Previous in vitro studies have reported that re-entry of G2/M -arrested cells into the cell cycle can lead to polyploidization, with DNA damage repair proteins such as ATM and ATR implicated in this process [63]. Cell-cycle reentry and subsequent endocycling of CE cells following UVA exposure was evident from EdU incorporation assays, with approximately 65% CE cells showing EdU positivity (indicative of new DNA synthesis). Furthermore, DAPI-based nuclear ploidy analysis, as previously described, revealed that endocycling initiated after day-2 and peaked at day-3 [9, 20, 21, 29, 48, 58, 64, 65]. By day-3, approximately 60% of surviving CE cells exhibited enhanced DNA content, with ploidy levels in some nuclei reaching as high as 64 C. Pretreating mice with the antioxidant NAC attenuated polyploidization, suggesting that endocycling was dependent on ROS. These findings are consistent with prior studies demonstrating that NAC pretreatment of mice reduces ROS production and mitigates oxidative stress-induced polyploidy in hepatocytes [38].

Since FECD predominantly affects females, with late-onset FECD having a greater incidence in women [4], we investigated how sex influences CE cell polyploidy following UVA-induced oxidative-DNA damage. Our results indicate that females exhibit a higher propensity for polyploidization than males. These findings are consistent with previous reports showing that estrogen promotes polyploidization of megakaryocytes through activation of estrogen receptor beta (ERβ) and upregulation of GATA1 [66]. Given that polyploidy promotes senescence and fibrosis (as discussed later), the greater susceptibility of women to FECD may, in part, be attributed to an increased capacity for polyploidization. Notably, polyploidization of CE cells was more prominent in the central cornea than in the periphery. This topographical difference may be due to higher UVA light absorption in the central cornea, a phenomenon commonly observed in FECD patients [56].

In our study, CE cell loss and subsequent polyploidization were accompanied by an increase in the size of the surviving cells. This inverse relationship between cell density and cell size suggests that surviving CE cells enhance ploidy to restore tissue mass, thereby maintaining CE integrity and tissue homeostasis during the early phase. This phenomenon has been observed in the Col8A2 mouse model of FECD, as well as in *Drosophila* tissues [21, 23]. Thus, equivalent tissue area/ volume is restored by the enhanced ploidy of fewer, yet larger cells. Indeed, polyploidy-associated cell growth has been shown to facilitate recovery following acute kidney injury [37] and lung injury [67]. Further studies are needed to elucidate the relationship between polyploidy and increased cell size, and to determine whether these features contribute to abnormal tissue repair responses in FECD.

We observed that, after reentering the cell cycle, CE cells rapidly endocycle until day-3, forming polyploid cells with an abnormally large nucleus. However, after day-7 they appeared to undergo endomitoses, generating multinucleated polyploid cells with smaller but multiple nuclei. Our EdU incorporation studies suggest that polyploidization (multinucleation) of CE cells is cell-cycle dependent. Our findings are consistent with a recent study [60] that reported ‘multinucleated giant cells’ with features resembling mitotic bodies in FECD patients following Descemet stripping only (DSO). However, in addition to endoreplication (endocycling and endomitosis), cells can also become polyploid through cell fusion, independent of the cell cycle [9]. In our study, cell boundaries of multinucleated polyploid cells were integral, as evidenced by ZO-1 staining. Nevertheless, the possibility that cell fusion contributes to the generation of multinucleated CE cells cannot be ruled out, and further studies are needed to clarify its role in FECD pathogenesis.

Polyploidy has been described as a stress response mechanism that enhances cell survival by providing additional time to withstand stress, resist apoptosis, and repair damaged DNA [13, 17]. In *Drosophila*, endoreplication silences p53-responsive genes, enabling polyploid cells to tolerate damage while resisting apoptosis [14, 58, 68]. A recent study by Chiara et al. have shown that polyploidy provides an acute survival advantage to tubular cells following acute kidney injury [37]. Since CE cells are normally non-dividing and replenishing them is challenging once lost, polyploidy may serve as a strategy for damaged CE cells to mitigate genomic damage following oxidative stress by having additional copies of newly synthesized DNA. This approach may provide extra time for DNA repair, thereby aiding in apoptosis resistance [15]. Further studies are needed to elucidate whether the initial tetraploid state of CE cells confers resistance to mild oxidative stress. Additionally, it remains to be determined if polyploidy provides a transient survival advantage to these cells in response to acute oxidative injury.

Accumulating evidence suggests cellular senescence plays a significant role in FECD [69], as senescence markers such as p21, p16, and p53 are upregulated in FECD patients [42, 43, 49, 70]. In vivo studies in aortic vascular smooth muscle cells revealed an increased expression of senescence markers (p16 and SA-β-gal) in polyploid cells [71]. Polyploidy induces senescence in cancer cells, enabling them to evade chemotherapy and radiation therapy [72, 73] as seen in CE cells in the early phase after UVA when cells are actively endocycling. However, in the later stage, the transition to endomitosis, resulting in multinucleated cells, correlates with the onset of senescence and subsequently fibrosis. These results align well with a previous study demonstrating that polyploidy promotes cellular senescence [74, 75] and interstitial fibrosis [37]. Due to their augmented genomic DNA content, which supports elevated transcriptional output and macromolecular secretion, polyploid cells exhibit a pro-secretory phenotype [28]. In FECD, senescent CE cells have been shown to produce a pro-fibrotic / pro-inflammatory secretome, known as the senescence-associated secretory phenotype (SASP), which negatively impacts the tissue microenvironment [43, 76].

Previous studies have demonstrated that YAP1, which controls the transcription of cell survival and cell-cycle genes, regulates endoreplication in mice and flies [14]. Hippo-YAP1 (or Yorkie in flies) signaling is involved in endocycling in Drosophila [21, 58, 77, 78], the liver [77, 79], and the kidney [37]. Our study demonstrates that YAP1 also regulates endocycling of CE cells following UVA exposure. After UVA exposure, we observed the nuclear translocation of YAP1, along with the upregulation of YAP1 mRNA and its downstream effector E2F1. This correlated with the upregulation of CDK2 and CDK1, and these effects were reversed by CA3, a YAP1 inhibitor. Our studies with human corneal endothelium samples showed the presence of multinucleated polyploid CE cells and significant upregulation of polyploidy markers (*YAP1, AKT1*, and *E2F1*) in FECD patient specimens, suggesting that YAP1 modulates CE cell polyploidy. Studies have shown that Hippo pathway inactivates YAP1 via cell contact inhibition [80] and loss of contact inhibition induces polyploidy [81]. Under normal conditions, the tightly packed honeycomb configuration of CE cells may provide necessary contact inhibition to prevent Hippo-YAP1 activation. However, UVA-induced cell loss may relieve contact inhibition, thereby activating YAP1 in CE cells and prompting them to polyploidize. Additional studies are necessary to determine whether oxidative stress activates YAP1 independently of contact inhibition [82].

Our study shows that YAP1 inhibition prevented polyploidization and biological consequences of chronic disease progression by decreasing hallmark FECD markers such as *ZEB1, COL8A2, COL4A1*, and *TGFBIp, FN-1*, *ACTA2, and CLU* seen in chronic disease progression; thus, providing a novel therapeutic target for FECD [43]. Importantly, YAP1 inhibition prevented CE cell hyperploidization and significantly rescued CE cell loss at day 14. While the difference between the UVA alone and the CA3 groups may appear modest at earlier time points, our quantitative analysis demonstrates a statistically significant rescue at day 14, with 19% preservation of CE cell density in the CA3-treated group. In the acute phase (day 1–7), CE cells undergo rapid cell-cycle reentry and endocycling, leading to polyploidization that initially serves as a compensatory, pro-survival response to UVA-induced stress. During this stage, cell density decline is primarily driven by acute injury, and inhibition of YAP1 has limited impact. However, beyond day 7, cells transition toward endomitosis, multinucleation, and subsequent senescence and fibrosis. It is during this phase (by day 14), when pathological changes become prominent, which means that YAP1 inhibition exerts a protective effect by preventing hyperploidization. This temporal pattern is consistent with our mechanistic findings showing that YAP1 regulates polyploidization and FECD-associated pathways. Therefore, the therapeutic effect of CA3 becomes more apparent at later stages when these processes are more established. Moreover, rescue of CE cell loss by inhibition of hyperploidization was also associated with a reduction in corneal edema, a functional read out of corneal endothelium affected by FECD. Our findings on rescue of UVA-induced CE loss by modifying hyperploidization align with previous reports showing that cell-cycle reentry and hyperploidization is a critical component of cell death in other post-mitotic cells, such as neurons [83]. Our data is further supported by other studies in which YAP1 inhibition ameliorated interstitial fibrosis in a chronic kidney disease model [37]. Based on the demonstrated efficacy in kidney disease [37], we adopted similar CA3 dose as a starting point for our mechanistic investigations. However, the optimal therapeutic dose for the treatment of FECD remains to be established. Particularly, critical parameters including ocular bioavailability, safety profile, and potential off-target effects need systematic evaluation in future studies.

To our knowledge, this is the first study that provide molecular insights into the role of polyploidy in FECD pathogenesis. Using our previously developed UVA-based mouse model of FECD, we characterized the temporal profile of ploidy changes in CE cells following oxidative stress. We further described the topographical difference in polyploidization of CE cells. Polyploidization is more robust in females than in males. Furthermore, immediately after oxidative stress, CE cells endocycle, forming polyploid cells, but subsequently, endomitosis generates multinucleated polyploid cells that are pro-senescent and pro-fibrotic. Finally, we demonstrate that CE cell polyploidization is mediated by YAP1 and that YAP1 inhibition rescues FECD progression in mice. Our study thus supports prevention of CE cell hyperploidization as a therapeutic strategy to prevent FECD.

## Methods

### Human tissue

This study was performed in accordance with the Declaration of Helsinki and was approved by the Institutional Review Board of Massachusetts Eye and Ear Infirmary. Written informed consent was obtained from patients undergoing surgical treatment for FECD. Post-keratoplasty specimens (de-identified) were obtained from surgeries performed at Massachusetts Eye and Ear (Boston, MA, USA) and PriceVision Group (Indianapolis, IN, USA). Normal donor corneas, collected and preserved in hypothermic storage media within 24 h of death, were obtained from Eversight Eye Bank (Ann Arbor, MI, USA) based on criteria reported in our previous studies [84–86]. Refer to Supplementary Table 1 for further details on human specimens used in this study.

### Animal study

This study utilized C57BL/6J wild-type mice obtained from either the Jackson Laboratory or Charles River. The mouse colonies were maintained in a climate-controlled animal care facility at Schepens Eye Research Institute, Boston, USA. Experimental mice were housed under a 12:12-h light/dark cycle with food and water available *ad libitum*. Mice were anesthetized with an intraperitoneal (i.p.) administration of ketamine (100 mg/kg) and xylazine (20 mg/kg). Animals were wrapped in heat retention drapes (SpaceDrapes, Inc., USA) during UVA exposure and in vivo imaging. All experimental procedures were approved by the Institutional Animal Care and Use Committee at Schepens Eye Research Institute and adhered to the ARVO Statement for the Use of Animals in Ophthalmic and Vision Research. The experiments were non-blinded and non-randomized however; the data analysis was blinded and was performed by two observers.

### Ultraviolet-A (UVA) light exposure model

UVA exposure of mouse eyes was performed as previously described [4]. Under ketamine/xylazine anesthesia, the right eye was exposed to UVA light, while the left eye served as an untreated control. A UVA LED light source (M365LP1; Thorlabs, USA; emission peak = 365 nm, FWHM bandwidth = 8 nm, irradiance = 398 mW/cm^2^) was focused on a 4 mm diameter illumination spot on the mouse cornea. The duration of UVA exposure (20 min 57 s) was adjusted to deliver the appropriate fluence (500 J/cm^2^), as measured with a thermal power sensor head (S425C, Thorlabs, USA) and energy meter console (PM100D, Thorlabs) [57].

### Immunostaining of whole-mount corneas

Mouse eyes were enucleated in sterile phosphate buffered saline (PBS), punctured in the posterior chamber using a 25-gauge needle, and fixed in 1 mL of 4% paraformaldehyde for 30 min. The corneal cup was then dissected and washed in a 24-well plate with 4 mL of 1X PBS for 15 min on an orbital shaker (60 rpm) at room temperature. The corneal cup was permeabilized in a 4 mL wash buffer (0.3% bovine serum albumin (BSA) + 0.3% Triton X-100 in 1X PBS) for 30 min on an orbital shaker (60 rpm) at room temperature, followed by blocking in a blocking buffer (2% BSA in 1X PBS) for 2 h at room temperature. Subsequently, the corneal cup was transferred to a 96-well plate and incubated with primary antibodies (Supplementary Table 2) in 200 μL of blocking buffer at 4 °C for 16 h on an orbital shaker (30 rpm). Afterward, the corneal cup was washed three times for 15 min each in 4 mL of wash buffer in a 24-well plate on an orbital shaker (60 rpm). It was then incubated with the corresponding secondary antibodies (Supplementary Table 2) for 2 h in the dark at room temperature. Following incubation with the secondary antibodies, DAPI (4′,6-diamidino-2-phenylindole) (stock solution 5 mg/mL in PBS) was added at a 1:1000 dilution and further incubated for 10 min at room temperature in the dark. The corneal cup was washed three additional times, each for 15 min, in 4 mL of wash buffer in the dark on an orbital shaker (60 rpm) at room temperature. Subsequently, the corneal cup was flattened with 3–4 radial cuts and mounted using DAPI-free mounting medium (H-1000; Vector Labs). A coverslip was applied and sealed with nail polish. Imaging was performed within 24 h using an SP8 confocal microscope with LAS-AF software [87].

### EdU labeling

Mice were injected with EdU (100 μg/20 g body weight) 1 h prior to, and on days 1 and 2 after UVA exposure. Corneas were harvested on day 3 post-UVA and fixed in 4% PFA for 1 h. EdU staining was performed according to the manufacturer’s instructions (Click-iT® EdU Imaging Kit, ThermoFisher, Catalog #C10338).

### Microscopy

Quantification of cells or substances of interest was conducted using four (200 × 200 μm) fields of view per cornea. One microscopic field was arbitrarily selected from each quarter of the cornea, corresponding to the regions marked by the radial cuts. The numbers obtained from the four fields were then averaged. Cell/nuclei counts and measurements of fluorescence intensities were performed by blinded observers using ImageJ software (U.S. National Institutes of Health, Bethesda, Maryland, USA).

### DAPI intensity-based ploidy calculation

DAPI and ZO-1 immunostaining of corneal cups were performed under consistent staining and imaging conditions. Four imaging areas were arbitrarily selected per cornea, one image from each quadrant marked by the radial cuts. DAPI intensity-based ploidy analysis was conducted as previously described [48]. For each microscopic field, full Z-stack images were captured using a Leica TCS SP5 confocal microscope with a 63× oil immersion objective, with a step size of 0.5 μm between slices for both DAPI and ZO-1 channels. The imaging settings were as follows: resolution 512 × 512, digital zoom = 1, smart gain = 703.4 V, smart offset = 0%, scan field rotation = 0, pinhole = 95.96 μm, zoom = 1. Images were saved as .tiff files, and Z-stack projections for each channel were created using the “Sum of Slices” function in ImageJ. In the DAPI channel Z-stack projection image, endothelial nuclei were magnified to 1600%, and each nucleus was manually outlined using the freehand drawing tool in ImageJ. These outlines were saved as Regions of Interest (ROIs). Overlapping nuclei and those touching the image border were excluded from analysis. The area and integrated density of each endothelial nucleus in the ROI map were measured using ImageJ’s analysis tools, and the values were exported to Microsoft Excel (Microsoft Corporation, 2018). To determine background fluorescence, three non-overlapping circles were drawn in different regions of the DAPI image using the circular selection tool. The area and integrated density of each background circle were also exported to the same Excel sheet. The average background per unit area was calculated by dividing each background integrated density by its corresponding area, then averaging the three values. The total background of each nucleus was determined by multiplying its area by the average background per unit area. This background value was subtracted from the measured integrated density to obtain the normalized DAPI intensity of each nucleus. For ploidy calculation, the normalized DAPI intensity of epithelial nuclei from unexposed eyes (representing a ploidy value of 2C) was used as a reference. The ploidy of each endothelial nucleus was calculated by dividing its normalized DAPI intensity by that of the reference epithelial nuclei, then multiplying by 2 to obtain the normalized ploidy (C-value) [48].

$${\rm{Endothelial\; Nuclear\; Ploidy}}=\frac{({\rm{Endothelial\; Nuclear\; Integrated\; density}}-{\rm{Background\; Nuclear\; Integrated\; density}})}{({\rm{Average\; Nuclear\; Integrated\; Density\; of\; unexposed\; epithelial\; nuclei}})}{\rm{x}}2$$

### Fibronectin-1 intensity quantification

Corneas were co-stained for Fibronectin-1 and ZO-1 as described above. Four images were acquired from each quadrant of the cornea using a SP8 confocal microscope with 63× oil objective. All staining and imaging parameters including exposure time, laser intensity, gain, and offset were kept identical for all images. Cell borders were delineated based on ZO-1 and DAPI staining to distinguish mononucleated and multinucleated cells. Cells touching the borders of the image were excluded. The fluorescence intensity of Fibronectin-1 immunostaining was measured by a blinded observer using ImageJ software (U.S. National Institutes of Health, Bethesda, Maryland, USA).

### In vivo imaging

Under ketamine/xylazine-induced anesthesia, the mouse was covered with thermally insulating drapes and positioned on a specialized platform designed to securely immobilize it for corneal endothelium (CE) imaging using a laser scanning in vivo confocal microscope equipped with the Heidelberg Retina Tomograph III (HRT III) and the Rostock Corneal Module (RCM) (Heidelberg Engineering). The confocal microscope captured 2D images representing cross-sectional views of the cornea, each measuring 400 × 400 μm (equivalent to 160,000 μm²) at a user-selectable corneal depth. These images were composed of 384 × 384 pixels and provided a lateral resolution of 1 μm/pixel. Digital images were stored on a computer workstation at a rate of 3 frames per second. To determine CE cell density in the HRT images, the Cell Counter plugin in ImageJ was used for analysis. CCT was measured using anterior segment optical coherence tomography (AS-OCT) with the Bioptigen Spectral Domain Ophthalmic Imaging System (Envisu R2200) equipped with a 12 mm telecentric lens (Bioptigen) to scan the cornea.

### Drug administration

N-acetylcysteine (Sigma-Aldrich, Catalog #A9165) was dissolved in normal saline and administered intraperitoneally at a dose of 1 g/kg body weight, 60 min prior to UVA exposure. CA3 (Selleckchem.com, Catalog #CIL56) was dissolved in 5% DMSO + 40% PEG300 + 5% Tween 80 + 50% ddH₂O, according to the manufacturer’s instructions. Control animals received an equivalent volume (100 μL) of the vehicle.

### Apoptosis assay

Apoptosis of mouse CE cells was detected using the Terminal Deoxynucleotidyl Transferase-Mediated Biotinylated UTP Nick End Labeling (TUNEL) assay (In Situ Cell Death Detection Kit, TMR Red, Sigma, Catalog #12156792910), following the manufacturer’s instructions. The enucleated eye was fixed in 4% paraformaldehyde for 30 min, after which the corneal cup was dissected, washed with 1× Phosphate Buffered Saline (PBS) three times for 15 min each, and incubated in the dark with 50 μL of TUNEL reaction mixture (5 μL of Enzyme Solution + 45 μL of Label Solution) for 60 min at 37 °C [88]. Subsequently, the corneal cup was counterstained with DAPI, washed with 1× PBS for 15 min, flattened with radial cuts, and mounted using DAPI-free mounting medium (H-1000; Vector Labs). Four regions of interest were arbitrarily selected in the central cornea (one from each quadrant) and Z-stack images were captured at ×40 magnification using an oil immersion objective with 1 μm intervals under an SP8 confocal microscope with LAS-AF software. The number of TUNEL-positive nuclei and total DAPI-positive nuclei in the Z-stack images were counted using the Cell Counter plugin in ImageJ.

### Quantitative RT-PCR

Corneal cups were harvested at different time points post-UVA and the corneal endothelium was stripped. RNA was extracted from the samples using Trizol (Invitrogen, Waltham, MA, USA), isolated and purified via sequential washes in absolute propanol and 75% ethanol and collected in nuclease-free water. The concentration of RNA was quantified using a NanoDrop instrument (ThermoFisher, Waltham, MA, USA). Complementary DNA (cDNA) was synthesized from the RNA using the iScript cDNA synthesis kit (Bio-Rad, Hercules, CA, USA) with a T100 Thermal cycler (Bio-Rad, Hercules, CA, USA) (temperature settings: 25 °C for 5 min, 46 °C for 20 min, and 95 °C for 1 min). RT-PCR was conducted using TaqMan gene expression assays (ThermoFisher, Waltham, MA, USA) in an Eppendorf Realplex2 epgradient S mastercycler (Eppendorf, Enfield, CT, USA). Results obtained were normalized to the GAPDH internal control, and relative expression was quantified as ΔΔ(−CT) [89].

### Periodic acid-schiff (PAS) staining

After enucleation, corneas were fixed in 4% paraformaldehyde (PFA) for 24 h, embedded in paraffin, and sectioned at a thickness of 7 μm using a microtome. For each eye, three coronal sections of the eye globe were taken from the center. Periodic acid–Schiff (PAS) staining was performed at the Morphology Core of the Schepens Eye Research Institute. Bright-field images were acquired at ×100 magnification using a Nikon Eclipse E800 microscope equipped with a DP70 camera. Three images were captured from arbitrarily selected areas in the central cornea for each section. The thickness of Descemet’s membrane was quantified by a blinded evaluator using ImageJ software (U.S. National Institutes of Health, Bethesda, Maryland, USA) [87].

### Statistics and reproducibility

Results are presented as mean ± standard error of the mean (SEM) with a 95% confidence interval, unless otherwise specified. Statistical analyses were performed using GraphPad Prism (version 9; GraphPad Software, La Jolla, California). Statistical significance was determined using one-way analysis of variance (ANOVA), followed by Tukey’s Honestly Significant Difference (HSD) post hoc test for multiple groups pairwise comparisons. Two-way ANOVA with Sidak’s test was used to analyze changes in CE cell density and CCT across different groups over multiple time points. Two-tailed Student’s t-tests were used for comparisons involving two groups. A *p*-value of <0.05 was considered statistically significant.

## Supplementary information

Supp. Table 1

Supp. Table 2

Supp. Figure 1

## Data Availability

The data generated during and/or analyzed during the current study is archived to Mass General Brigham (MGB) Dropbox with unlimited storage and Schepens Eye Research Institute of Mass Eye and Ear server. The data is available upon request from the corresponding author.
