# Supplementary material for: Ultraviolet-A light-induced chronic hyperploidization causes fibrosis in post-mitotic cells affected by Fuchs endothelial corneal dystrophy
Source: Cell Death Discov. 2026 Aug 8;12:442. doi: 10.1038/s41420-026-03292-8 (PMC13736856; doi:10.1038/s41420-026-03292-8)
Supplement: Supplementary file 1 [file 41420_2026_3292_MOESM3_ESM.pdf]

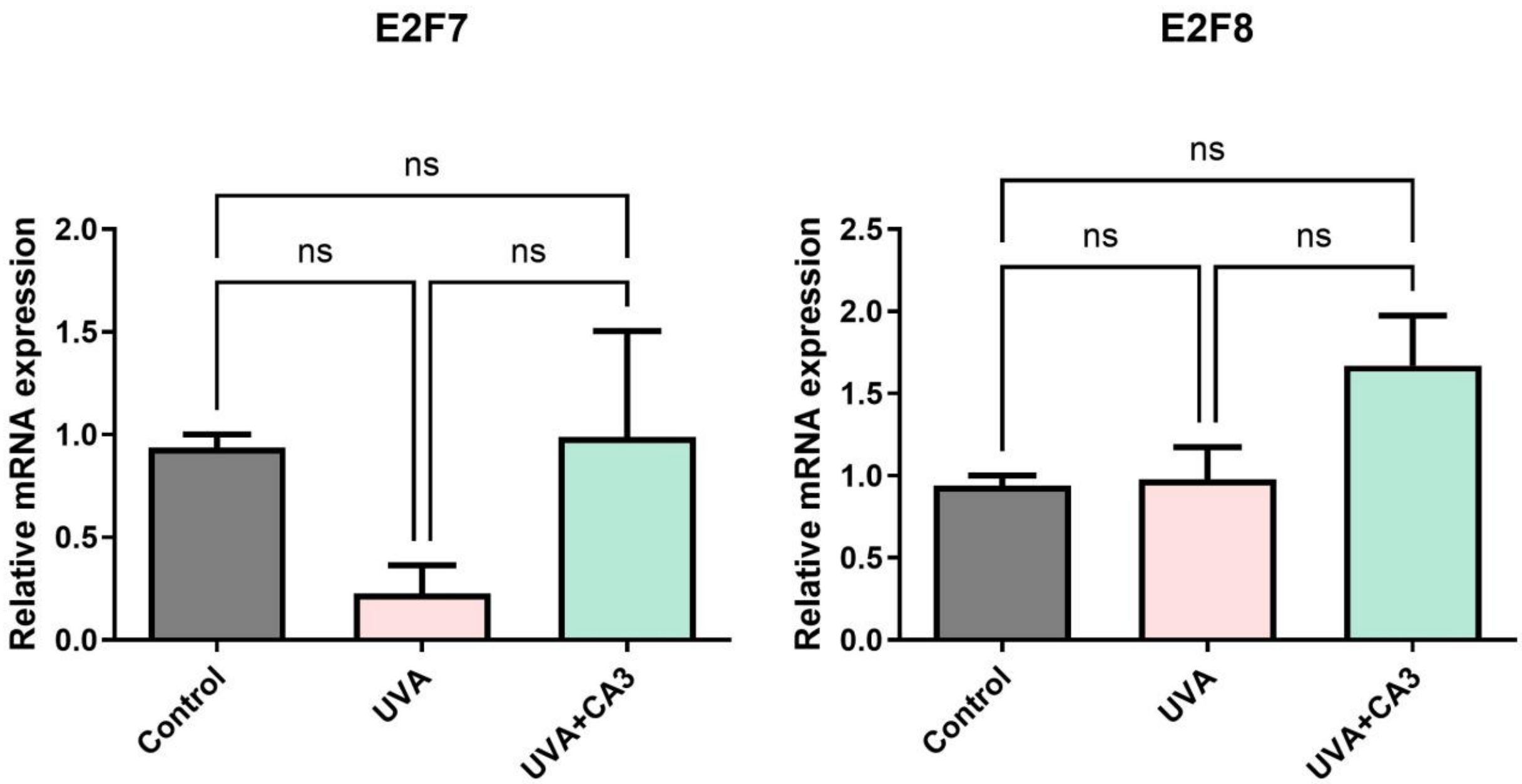

Supplementary Fig. 1. RT-PCR data showing E2F7 and E2F8 not elevated in the UVA or UVA+CA3 groups. n=3 biological replicates. ns=not significant.
