# Supplementary material for: Ultraviolet-A light-induced chronic hyperploidization causes fibrosis in post-mitotic cells affected by Fuchs endothelial corneal dystrophy
Source: Cell Death Discov. 2026 Aug 8;12:442. doi: 10.1038/s41420-026-03292-8 (PMC13736856; doi:10.1038/s41420-026-03292-8)
Supplement: Supplementary file 2 [file 41420_2026_3292_MOESM1_ESM.docx]

| **Supplementary Table 1.** Details and characteristics of human donor corneas and FECD patient specimens used in this study. | | | | |
| --- | --- | --- | --- | --- |
| **Cornea donor information** | | | | |
| **Donor ID** | **Age (years)** | **Death to preservation (hours)** | **ECD (cells/mm^2^)** | **Sex** |
| Control 1 | 64 | 13.33 | 3247 | F |
| Control 2 | 56 | 24.38 | 2762 | M |
| Control 3 | 71 | 5.31 | 2597 | M |
| Control 4 | 62 | 13.33 | 3236 | F |
| Control 5 | 63 | 13.34 | 1862 | M |
| Control 6 | 75 | 5.30 | 1912 | F |
|  |  |  |  |  |
|  | **Age** | **Surgery to utilization (Days)** | **ECD** | **Sex** |
| FECD 1 | 78 | 5 | N/A | F |
| FECD 2 | 50 | 5 | N/A | F |
| FECD 3 | 74 | 3 | N/A | M |
| FECD 4 | 78 | 5 | N/A | F |
| FECD 5 | 77 | 1 | N/A | F |
| FECD 6 | 86 | 1 | N/A | F |
| FECD 7 | 73 | 1 | N/A | M |
| FECD 8 | 75 | 1 | N/A | F |
|  |  |  |  |  |
