# Supplementary material for: Ultraviolet-A light-induced chronic hyperploidization causes fibrosis in post-mitotic cells affected by Fuchs endothelial corneal dystrophy
Source: Cell Death Discov. 2026 Aug 8;12:442. doi: 10.1038/s41420-026-03292-8 (PMC13736856; doi:10.1038/s41420-026-03292-8)
Supplement: Supplementary file 3 [file 41420_2026_3292_MOESM2_ESM.docx]

**Supplementary table 2*.*** List of reagents and resources used in this study with the catalogue number or specific identifier.

| **REAGENT or RESOURCE** | **SOURCE** | **IDENTIFIER** |
| --- | --- | --- |
|  | | |
| Mice | Charles River | C57BL/6J |
| Instruments | | |
| UVA light source | Thorlabs | Model #M365LP1 |
| Power sensor | Thorlabs | Model #S425C |
| Heidelberg Retina Tomograph III | Heidelberg engineering | with RCM module |
| Anterior segment–optical coherence tomography | Bioptigen | with Envisu software |
| SP8 confocal | Leica microsystems |  |
| SP5 confocal | Leica microsystems |  |
| NanoDrop™ 2000 | Thermo Scientific | ™ 2000 |
| Primary antibodies | | |
| Mouse anti-ZO-1 monoclonal (1:200) | ThermoFisher | Cat#339100 |
| Rabbit anti-Ki67 polyclonal | Abcam | Ab15580 |
| Rabbit anti-FN-1 monoclonal | Abcam | Ab2413 |
| Rabbit anti-YAP1 monoclonal | Abcam | Ab205270 |
| Rabbit anti-H3K9me3 monoclonal | Sigma | PA531910 |
| Secondary antibodies | | |
| Goat anti-mouse – FITC (1:500) | Jackson | 115-095-003 |
| Donkey anti-Rabbit Rhodamine (1:500) | Jackson | 711-295-152 |
| Chemicals and Critical commercial assays | | |
| Click-iT™ EdU Cell Proliferation Kit | ThermoFisher | C10339 |
| In Situ Cell Death Detection Kit, TMR red (TUNEL) | Sigma | 12156792910 |
| 4′,6-Diamidino-2-phenylindole dihydrochloride (DAPI) | Millipore Sigma | D9542 |
| Paraformaldehyde (16%) | Electron microscopy sciences | SKU 15610-S |
| Phosphate buffered saline (10X, pH 7.4) | Boston Bioproducts | BM-220-10XS |
| Bovine Serum Albumin | Fisher Scientific | BP1600-100 |
| Anti-Fade Mounting medium without DAPI | Vectashield | H-1900-10 |
| Trizol | Life technologies | 15596018 |
| Isopropanol | Fisher Scientific | LC157502 |
| Chloroform | Sigma | 372978 |
| iScript cDNA synthesis kit | Bio-rad | 1708891 |
| N-Acetyl-L-cysteine | Millipore sigma | A9165 |
| Dimethyl sulfoxide | Millipore sigma | D8418 |
| Polyethylene glycol | Millipore sigma | 81172 |
| Tween 80 | Millipore sigma | P1379 |
| CA3 | Selleckchem | S8661 |
| Primers | | |
| Mouse p53 | Life technologies | Mm01731290_g1 |
| Mouse CDK1 | Life technologies | Mm00772472_m1 |
| Mouse CDK2 | Life technologies | Mm00443947_m1 |
| Mouse CDK4 | Life technologies | Mm00677718_g1 |
| Mouse CDK6 | Life technologies | Mm01311342_m1 |
| Mouse YAP1 | Life technologies | Mm01143263_m1 |
| Mouse AKT1 | Life technologies | Mm01331626_m1 |
| Mouse E2F1 | Life technologies | Mm00432939_m1 |
| Mouse E2F7 | Life technologies | Mm00618098_m1 |
| Mouse E2F8 | Life technologies | Mm01204160_m1 |
| Mouse CDKN2A | Life technologies | Mm00494449_m1 |
| Mouse CDKN2A | Life technologies | Mm00432448_m1 |
| Mouse ZEB1 | Life technologies | Mm00495564_m1 |
| Mouse SNAI1 | Life technologies | Mm00441533_g1 |
| Mouse SNAI2 | Life technologies | Mm00441531_m1 |
| Mouse ACTA2 | Life technologies | Mm00725412_s1 |
| Mouse FN1 | Life technologies | Mm01256744_m1 |
| Mouse COL8A2 | Life technologies | Mm02344867_g1 |
| Mouse COL4A1 | Life technologies | Mm01210125_m1 |
| Mouse TGFβIP | Life technologies | Mm01337605_m1 |
| Mouse CLU | Life technologies | Mm01197002_m1 |
| Mouse GAPDH | Life technologies | Mm99999915_g1 |
| Human YAP1 | Life technologies | Hs00902712_g1 |
| Human AKT1 | Life technologies | Hs00178289_m1 |
| Human E2F1 | Life technologies | Hs00153451_m1 |
| Human GAPDH | Life technologies | Hs99999905_m1 |
| Software | | |
| ImageJ | National Institutes of Health (NIH) |  |
| LAS-AF | Leica Microsystems |  |
|  | | |
